# Establishing an Analogue Based In Silico Pipeline in the Pursuit of Novel Inhibitory Scaffolds against the SARS Coronavirus 2 Papain-Like Protease

**DOI:** 10.3390/molecules26041134

**Published:** 2021-02-20

**Authors:** Roxanna Hajbabaie, Matthew T. Harper, Taufiq Rahman

**Affiliations:** Department of Pharmacology, Cambridge University, Tennis Court Road, Cambridge CB2 1PD, UK; rh731@cam.ac.uk (R.H.); mth29@cam.ac.uk (M.T.H.)

**Keywords:** drug discovery, papain-like protease, PL^pro^, SARS coronavirus 2, COVID-19, analogues, in silico, docking, scaffold hopping, virtual screening

## Abstract

The ongoing coronavirus pandemic has been a burden on the worldwide population, with mass fatalities and devastating socioeconomic consequences. It has particularly drawn attention to the lack of approved small-molecule drugs to inhibit SARS coronaviruses. Importantly, lessons learned from the SARS outbreak of 2002–2004, caused by severe acute respiratory syndrome coronavirus 1 (SARS-CoV-1), can be applied to current drug discovery ventures. SARS-CoV-1 and SARS-CoV-2 both possess two cysteine proteases, the main protease (M^pro^) and the papain-like protease (PL^pro^), which play a significant role in facilitating viral replication, and are important drug targets. The non-covalent inhibitor, GRL-0617, which was found to inhibit replication of SARS-CoV-1, and more recently SARS-CoV-2, is the only PL^pro^ inhibitor co-crystallised with the recently solved SARS-CoV-2 PL^pro^ crystal structure. Therefore, the GRL-0617 structural template and pharmacophore features are instrumental in the design and development of more potent PL^pro^ inhibitors. In this work, we conducted scaffold hopping using GRL-0617 as a reference to screen over 339,000 ligands in the chemical space using the ChemDiv, MayBridge, and Enamine screening libraries. Twenty-four distinct scaffolds with structural and electrostatic similarity to GRL-0617 were obtained. These proceeded to molecular docking against PL^pro^ using the AutoDock tools. Of two compounds that showed the most favourable predicted binding affinities to the target site, as well as comparable protein-ligand interactions to GRL-0617, one was chosen for further analogue-based work. Twenty-seven analogues of this compound were further docked against the PL^pro^, which resulted in two additional hits with promising docking profiles. Our in silico pipeline consisted of an integrative four-step approach: (1) ligand-based virtual screening (scaffold-hopping), (2) molecular docking, (3) an analogue search, and, (4) evaluation of scaffold drug-likeness, to identify promising scaffolds and eliminate those with undesirable properties. Overall, we present four novel, and lipophilic, scaffolds obtained from an exhaustive search of diverse and uncharted regions of chemical space, which may be further explored in vitro through structure-activity relationship (SAR) studies in the search for more potent inhibitors. Furthermore, these scaffolds were predicted to have fewer off-target interactions than GRL-0617. Lastly, to our knowledge, this work contains the largest ligand-based virtual screen performed against GRL-0617.

## 1. Introduction

As one of the greatest health crises of our generation, the ongoing coronavirus disease 2019 (COVID-19) pandemic has resulted in more than 2 million deaths, with approximately 110 million cases reported worldwide [1], and profound socioeconomic impact [2,3,4,5]. Severe acute respiratory syndrome coronavirus 2 (SARS-CoV-2) [6], the causative agent of COVID-19 [7], is the seventh known coronavirus to infect humans [8]. SARS-CoV-2 is highly infectious [9,10,11,12], and spreads through particles expelled from infected individuals [13,14]. As the most severe pandemic caused by a coronavirus [15], COVID-19 has caused more cases and fatalities than the severe acute respiratory syndrome (SARS) pandemic, and the Middle East respiratory syndrome (MERS)-related outbreaks, combined [16]. In 2015, SARS and MERS were listed by the World Health Organization (WHO) as likely to be the cause of a future epidemic or pandemic and requiring urgent research and development (R&D) [17]. In December 2020, the Pfizer-BioNTech COVID-19 vaccine [18,19], and the Moderna COVID-19 vaccine [20,21] were approved by the United States Food and Drug Administration (FDA), and the Oxford-AstraZeneca COVID-19 vaccine [22,23,24] was approved by the United Kingdom Medicines and Healthcare products Regulatory Agency (MHRA). Other vaccines have also been authorized for emergency use, including the Sputnik V COVID-19 vaccine (Russia) [25] and CoronaVac (China) [26]. As the first vaccines approved for the prevention of COVID-19, they have offered a renewed picture of hope towards the end of the pandemic.

However, given the evolving nature of the virus, as well as high mortality rates, approved small-molecule drugs for COVID-19 remain highly sought-after. SARS-CoV-2 has several important drug targets. These include the main protease (M^pro^) [27,28], and the papain-like protease (PL^pro^). PL^pro^’s name is derived from its structural similarity to papain, an enzyme found in papaya. There is currently no approved drug that acts against the M^pro^ or PL^pro^. Remdesivir, which has been approved by the FDA as the first and only small-molecule drug for the treatment COVID-19 in hospitalized patients [29], is suggested to target the SARS-CoV-2’s RNA-dependent RNA polymerase (RdRp) [30,31]. However, there are conflicting studies regarding the efficacy of remdesivir in the patient population [32,33,34,35]. Furthermore, it cannot be administered orally. Thus, the search for a highly potent and specific antiviral continues.

The M^pro^ has been the subject of intense scrutiny, with 23 investigational inhibitors of varying potency identified thus far [36,37]. Conversely, only one small-molecule inhibitor, GRL-0617, with experimental data has been reported for the SARS-CoV-2 PL^pro^, by the International Union of Basic and Clinical Pharmacology and the British Pharmacological Society (IUPHAR/BPS) Guide to Pharmacology [36,37], with a half-maximal inhibitory concentration (IC_50_) of 2.4 µM [38]. Ebselen has also been presented as a potential inhibitor of PL^pro^ in a pre-print article [39]. GRL-0617 binds non-covalently, and it is the only PL^pro^ inhibitor with a co-crystallised structure [40] available in the Protein DataBank (www.rcsb.org) (PDB ID: 7JRN). This co-crystallised structure can serve as a valuable tool to analyse the binding mode of GRL-0617, which can aid drug discovery efforts. GRL-0617 was initially found to inhibit the SARS-CoV-1 PL^pro^ in 2008, with an IC_50_ of 600 nM [41]. The SARS-CoV-1 and SARS-CoV-2 PL^pro^’s share 83% sequence identity and are structurally similar [42,43]. Although GRL-0617 demonstrates good potency, there is a lack of data on its pharmacokinetic profile. Furthermore, GRL-0617 contains a toxic chemical moiety, an aniline group. Nearly one-third of drugs that have been withdrawn from the market, or that have black-box warnings associated with idiosyncratic adverse drug reactions (IADRs), contain an aniline group [44,45,46,47]. GRL-0617 has not yet been tested in clinical trials, or in animal studies, to establish safety or side-effects. The presence of other inhibitory scaffolds may, therefore, be useful to aid and accelerate the drug discovery process, as safety concerns account for a major reason candidate drugs fail to be marketed after phase 3 trials [48,49].

**Figure 1:**
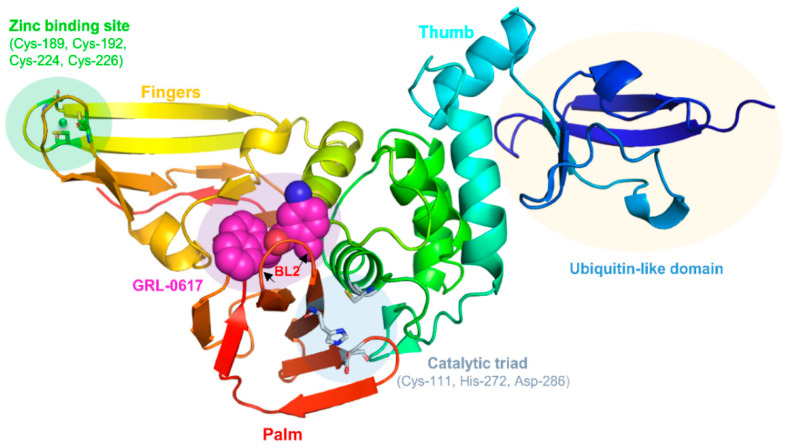
The structure of PL^pro^ (PDB ID: 7JRN). The ubiquitin-binding domain is shown in dark blue. The catalytic triad is shown as silver sticks. Bound GRL-0617 is shown as magenta spheres, with BL2 (red) labelled nearby. The zinc-binding site is coordinated by residues shown as green sticks. The zinc ion is shown as a green sphere. In general, domain 1: dark blue, domain 2 (‘thumb’): green/cyan, domain 3 (‘fingers’): yellow/orange, domain 4 (‘palm’): red.

Upon entry of SARS-CoV-2 into human cells, the M^pro^ and PL^pro^ play a major role in aiding the viral replication process [50,51]. They are cysteine proteases, responsible for processing the replicase polyproteins, polyprotein 1a (pp1a), and polyprotein 1ab (pp1ab), which are derived from the RdRp. This facilitates the generation of 16 products, non-structural proteins (NSPs) 1 to 16. PL^pro^, which itself is encoded in NSP 3, cleaves NSPs 1-3, whilst the M^pro^ cleaves NSPs 4-16 [52]. The NSPs play an instrumental role in initiating viral replication. Targeting the M^pro^ or PL^pro^ with inhibitory small-molecules will, therefore, halt viral replication. As shown in Figure 1, the recently solved crystal structure of PL^pro^ (PDB ID: 7JRN) [40,42,53] is composed of four domains: a ubiquitin-like domain, a catalytic (‘thumb’) domain, a zinc-binding (‘fingers’) domain, and a fourth (‘palm’) domain, which contributes two residues to the catalytic triad. Unlike the M^pro^, the SARS-CoV-2 PL^pro^ can cleave interferon-stimulated gene 15 (ISG15) in the cytosol of host cells [38,54,55,56]. This causes dysregulation of signalling cascades and can lead to a ‘cytokine storm’, which is associated with increased severity of morbidity, as well as an increased likelihood of mortality, in COVID-19 patients [57].

Importantly, a stabilising mutation at NSP 3 may suggest a mechanism which differentiates COVID-19 from SARS [58,59]. NSP 3 has a major role in suppressing the host’s innate immunity, and is associated with the inflammation produced in severe COVID-19. Furthermore, the increased infectivity of SARS-CoV-2, in contrast to SARS-CoV-1, may be related to a destabilising mutation at NSP 2 [58,59]. Interestingly, the PL^pro^ of SARS-CoV-1 preferentially cleaves ubiquitin over ISG15 [60]. The catalytic site of PL^pro^ harbours the catalytic triad (Figure 1): Cys-111, His-272, and Asp-286 [50,61]. The known non-covalent inhibitor GRL-0617 does not directly form contacts with the catalytic triad, but instead, it binds to a cavity nearby and induces the closure of blocking loop 2 (BL2) [53,62]. This results in a narrowing of the catalytic site, preventing substrate binding, and catalysis. The zinc-binding site is coordinated by the conserved cysteines, Cys-189, Cys-192, Cys-224, and Cys-226. Zinc binding plays a significant role in the structural integrity of the protein [50,61].

In this work, we used GRL-0617 as a reference molecule to virtually screen over 339,000 lead-like and diverse molecules in the chemical space. This was followed by various rounds of docking against the PL^pro^ crystal structure, as well as evaluation of drug-likeness, to search for lead scaffolds against PL^pro^. In preparation for future coronavirus pandemics, the presence of such scaffolds in the literature may expand our toolkit and inform drug design, development, and discovery efforts, in the search for potent inhibitors. To prevent the next coronavirus epidemic from becoming a pandemic of such a grand scale, an approved drug against SARS-CoV-2 may be repurposed for immediate use. This will also preclude the need to create a new vaccine, which is a highly time-consuming process.

Although a small-molecule drug that targets both M^pro^ and PL^pro^ is near impossible to design, it may be possible to find potent inhibitors for each of the proteases separately but administer them in combination. A drug that inhibits both proteases responsible for processing the viral polyprotein replicases may be more effective than a drug that inhibits one of the proteins. Importantly, such molecules should be lipophilic to penetrate the plasma membrane of host cells and reach the M^pro^ or PL^pro^. Here, we present several novel drug-like, and lipophilic scaffolds with structural and electrostatic similarity to GRL-0617, and comparable protein-ligand interactions. These scaffolds may be tested in vitro to establish effectiveness in comparison to GRL-0617 and, if proven to be active, their efficacy can be further improved through structure-activity relationship (SAR) studies.

## 2. Results

### 2.1. Round 1: Ligand-Based Virtual Screening

**Figure 2:**
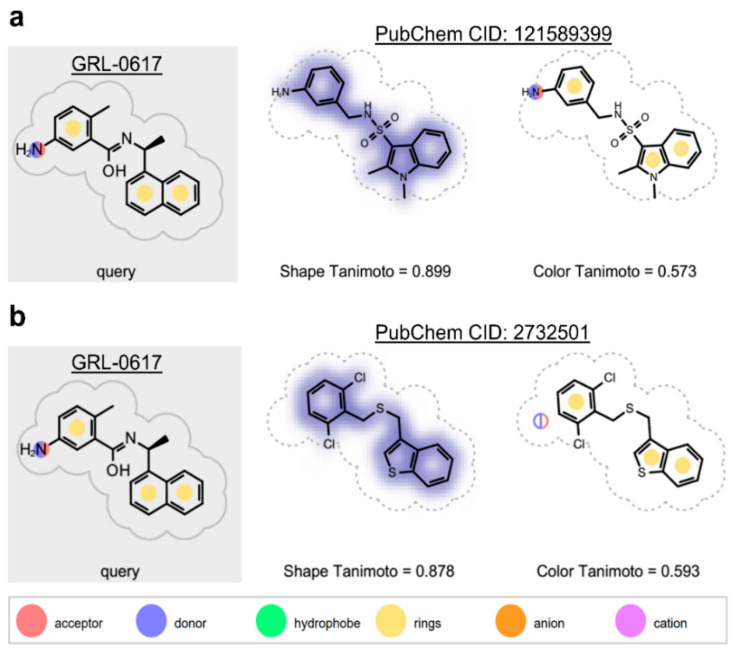
ROCS results (highest scoring molecules). (**a**) PubChem CID 121589399 had obtained the highest Shape Tanimoto; (**b**) PubChem CID 2732501 had obtained the highest Color Tanimoto.

**Figure 3:**
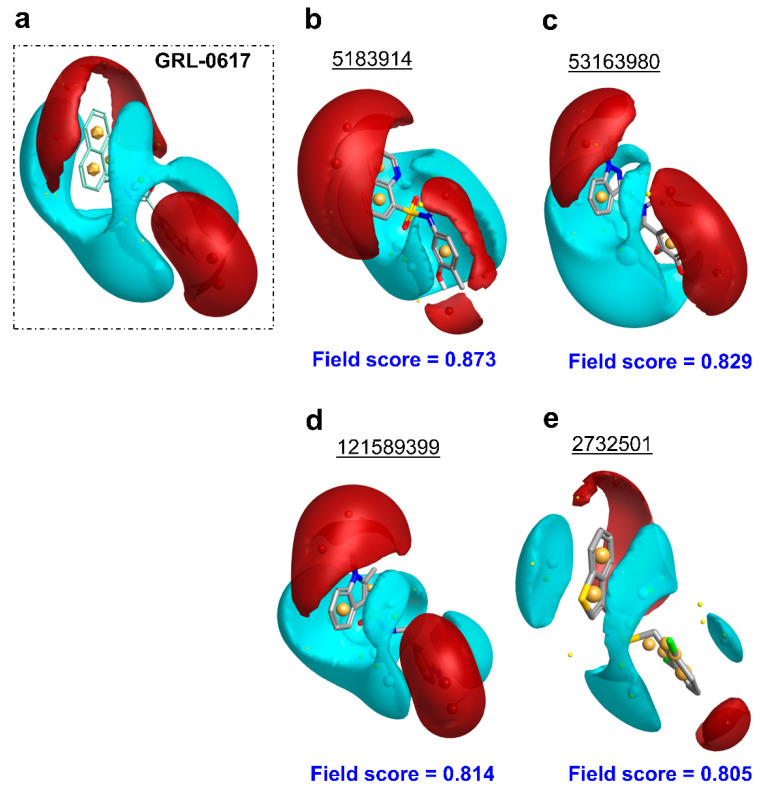
Forge results of the compounds’ electrostatic similarity to GRL-0617 (hits scoring above 0.8 shown). (**a**) Query molecule GRL-0617 (shown as light green lines), which all compounds were aligned to; (**b**) Compound with PubChem CID: 5183914; (**c**) Compound with PubChem CID 53163980; (**d**) Compound with PubChem CID 121589399; (**e**) Compound with PubChem CID 2732501. Field scores for each of the compounds are shown in blue font. Regions of positive electrostatic potential are shown in red. Regions of negative electrostatic potential are shown in cyan. Regions of hydrophobicity are shown as gold spheres. The hits are shown as grey sticks.

A screen of 339,240 molecules from the ChemDiv Diversity^®^, MayBridge Hit Locator^®^, and Enamine Hit Locator^®^ chemical libraries in Rapid Overlay of Chemical Structures (ROCS) (OpenEye Scientific Software, Santa Fe, NM, USA) [63,64] and subsequent electrostatic field comparison in Forge (Cresset, Litlington, Cambridgeshire) [65,66,67], revealed 24 similar hits to the reference ligand, GRL-0617 (Supporting Information, Figure S1), which were commercially available (Supporting Information, Table S1). Of these hits, 18 had a Shape Tanimoto score (denoting structural/3D shape similarity to the reference) above 0.8 (Supporting Information, Figure S2), with the highest being that of the hit with PubChem Compound ID (CID) 121589399 (Figure 2a). Six hits obtained a Color Tanimoto score (denoting chemical group similarity to reference) above 0.5, with the highest being that of the hit with PubChem CID 2732501 (Figure 2b). Importantly, four compounds (Figure 3b–e; Supporting Information, Figure S3), including 121589399 obtained field scores (denoting electrostatic similarity to reference) above 0.8. Hits with field scores of 0.8 and above have a strong potential to be bioactive [67]. 

### 2.2. Validation of the Docking Protocol

**Figure 4:**
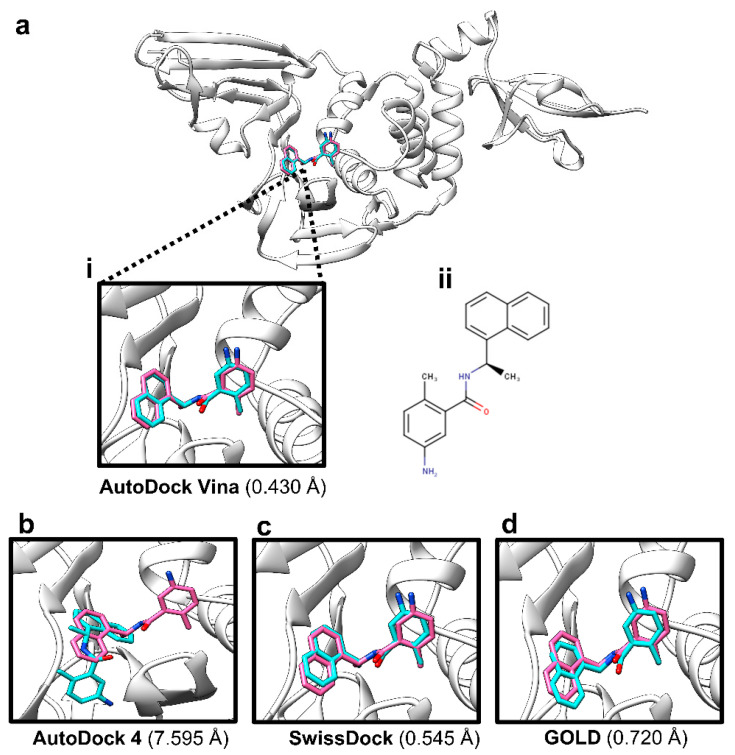
Validation of the docking protocol using GRL-0617. (**a**)-**i** Reproducing the co-crystallised pose of GRL-0617 in AutoDock Vina (co-crystallised pose: pink sticks, docked pose: cyan sticks), with an RMSD of 0.430 Å between the two poses. The protein crystal structure is shown as white ribbons (PDB ID: 7JRN); (**a**)-**ii** The two-dimensional structure of GRL-0617 (drawn in MarvinSketch); (**b**) Co-crystallised pose: pink sticks, docked pose in AutoDock 4.2: cyan sticks, with an RMSD of 7.595 Å between the two poses; (**c**) Co-crystallised pose: pink sticks, docked pose in SwissDock: cyan sticks, with an RMSD of 0.545 Å between the two poses; (**d**) Co-crystallised pose: pink sticks, docked pose in GOLD: cyan sticks, with an RMSD of 0.720 Å between the two poses.

Blind docking [67,68] of the reference ligand GRL-0617 against PL^pro^ in several programs showed that AutoDock Vina [69] could best reproduce the co-crystallised GRL-0617 pose, as shown in Figure 4. When the docked pose was superimposed against the co-crystallised pose, there was found to be a root-mean-square-deviation (RMSD) of ~0.4 Å (within the ≤2 Å acceptable range for validation of self-docking). Furthermore, the score and pose were highly reproducible in five independent docking runs. The mean predicted free energy of binding (Gibbs free energy (∆G)) score, which is often reported as the predicted binding affinity in docking, calculated for GRL-0617 was −9.68 ± 0.22 kcal/mol (mean ± standard error of the mean (SEM)), for five independent runs (Supporting Information, Table S2).

### 2.3. Evaluation of Hits from Virtual Screening Using Molecular Docking

#### 2.3.1. Round 2: Docking 24 Hits from ROCS

**Figure 5:**
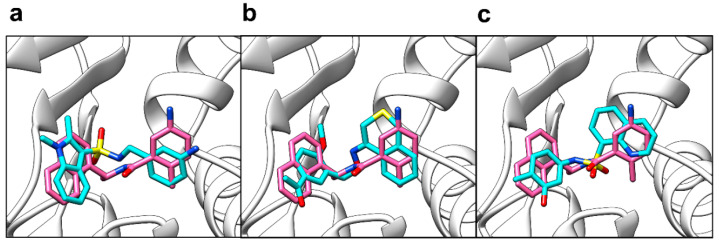
Poses of compounds from blind docking against PL^pro^ (PDB ID: 7JRN) in AutoDock Vina. (**a**) Compound 121589399 pose (cyan sticks) shown relative to reference GRL-0617 co-crystallised pose (pink sticks); (**b**) Compound 5384279 pose (cyan sticks) shown relative to reference GRL-0617 co-crystallised pose (pink sticks); (**c**) Compound 5183914 (cyan sticks) shown relative to reference co-crystallised GRL-0617 pose (pink sticks). The protein crystal structure is shown as white ribbons.

The 24 distinct chemical scaffolds obtained from virtual screening were then blindly docked against PL^pro^ in AutoDock Vina (Scripps Research, San Diego, CA, USA) [69] (Supporting Information, Table S2). Of these, compounds with poses that were not in the active site in any of the five runs were automatically eliminated from the search (PubChem CIDs: 46295452, 4818469, 5079298, 135431927, 20878059). The compound with PubChem CID 121589399 obtained the best (most negative) mean predicted binding affinity of −8.88 ± 0.07 kcal/mol (*n* = 5), with its pose in the same site as GRL-0617 in all five runs (Figure 5a). This compound also obtained the best Shape Tanimoto score in the ligand-based screening. Additionally, it was one of the hits with the highest field score (0.814). PubChem CID 5384279 obtained the second-best mean predicted binding affinity of −8.70 ± 0.05 kcal/mol (*n* = 5), with its pose in the same site as GRL-0617 in all five runs (Figure 5b). The compound with PubChem CID 5183914, which notably obtained the highest field score of 0.873, had a mean predicted binding affinity of −8.44 ± 0.14 kcal/mol (*n* = 5), with its pose in the same site as GRL-0617 in all five runs (Figure 5c). 

**Figure 6:**
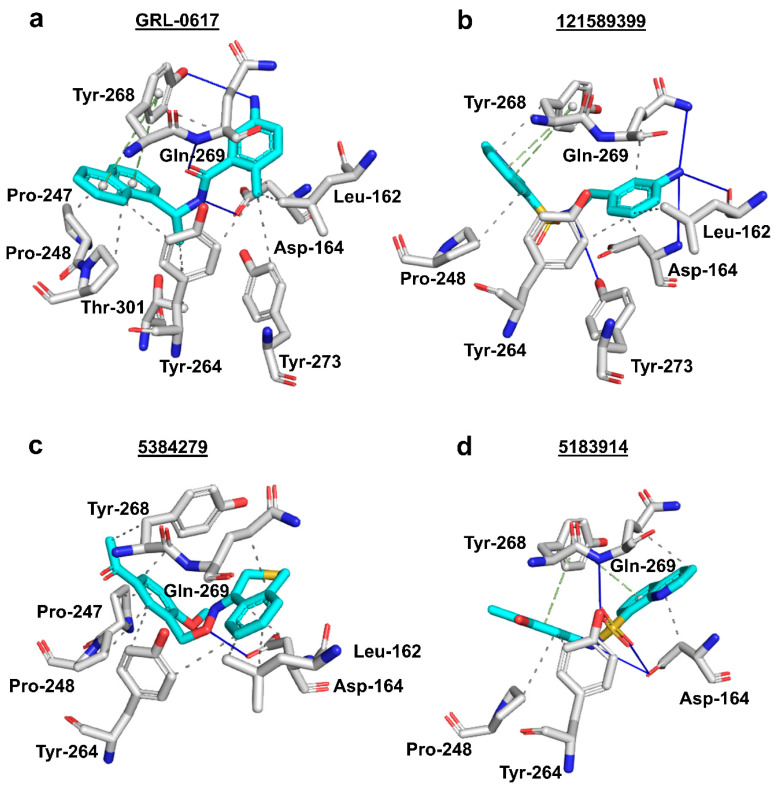
Three-dimensional protein-ligand interactions predicted by PLIP for the reference and three hits. (**a**) Reference ligand GRL-0617’s (cyan sticks) interactions with PL^pro^ residues (white/silver sticks); (**b**) Compound 121589399’s (cyan sticks) interactions with PL^pro^ residues (white/silver sticks); (**c)**) Compound 5384279’s (cyan sticks) interactions with PL^pro^ residues (white/silver sticks); (**d**) Compound 5183914’s (cyan sticks) interactions with PL^pro^ residues (white/silver sticks). 3-letter codes and sequence numbers for amino acids are given. Hydrogen bonds are shown as blue lines. π-π stacking interactions are shown as green dashes. Hydrophobic interactions are shown as grey dots. The PubChem CID is shown above each compound.

Three-dimensional analysis of the 24 docked compounds’ protein-ligand interactions in Protein-Ligand Interaction Profiler (PLIP) (BIOTEC, Tatzberg, Dresden) [70] revealed that compounds with PubChem CID 121589399 and PubChem CID 5384279 had the greatest number of interactions in common with the reference ligand (Figure 6a–c). For compound 121589399, these were: π-π stacking with Tyr-268, hydrogen bonds with Leu-162, Asp-164, Gln-269, and Tyr-273, as well as hydrophobic interactions with Leu-162, Asp-164, Pro-248, Tyr-264, and Tyr-268, and Gln-269. Although compound 5384279 was missing the π-π stacking with Tyr-268, it had retained the hydrogen bond with Asp-164, and hydrophobic interactions with Leu-162, Asp-164, Pro-247, Pro-248, Tyr-264, Tyr-268, and Gln-269.

**Figure 7:**
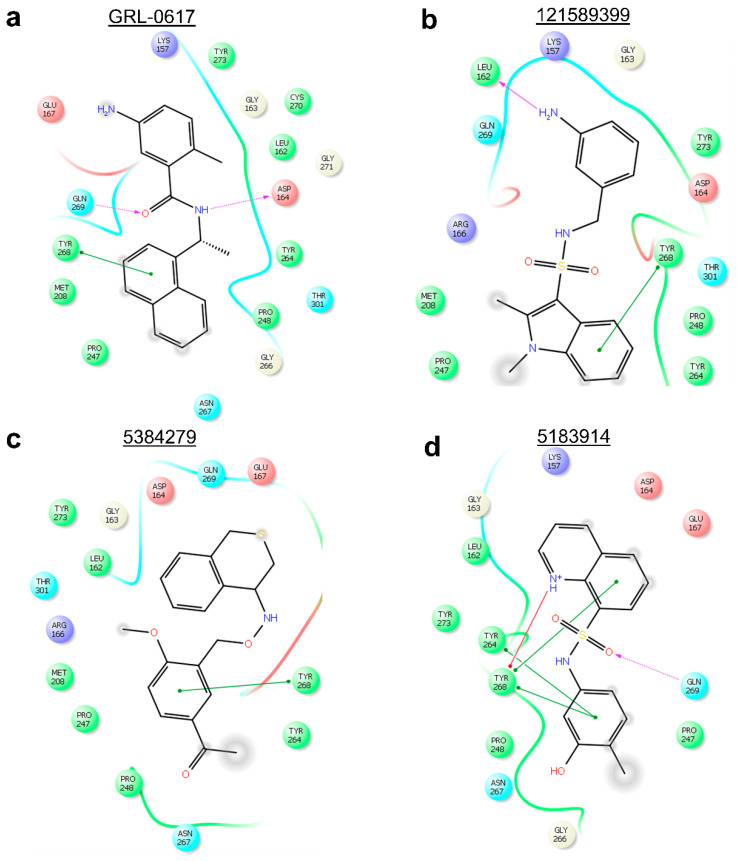
Two-dimensional protein-ligand interactions predicted by Maestro for the reference and three hits. (**a**) Reference ligand GRL-0617’s interactions with PL^pro^ residues; (**b**) Compound 121589399’s interactions with PL^pro^ residues; (**c**) Compound 5384279’s interactions with PL^pro^ residues; (**d**) Compound 5183914’s interactions with PL^pro^ residues. π-π stacking interactions are represented by green lines. π-cation interactions are represented by red lines. Hydrogen bonds are represented by purple dashed arrows (side chain) and purple solid arrow (backbone). Hydrophobic residues are shown as green circles. Positively charged residues are shown as purple circles. Negatively charged residues are shown as red circles. Polar residues are shown as cyan circles. Glycines are shown as light-yellow circles. The PubChem CID is shown above each compound.

Two-dimensional analysis of these two compounds’ interactions in Maestro (Schrödinger, L.L.C., New York, NY, USA) [71] showed that both compounds formed π-π stacking with Tyr-268, like the reference ligand (Figure 7a–c). Analysis of compound 5183914’s interactions in PLIP (Figure 6d) revealed several residue interactions in common with the reference ligand: π-π stacking with Tyr-268, hydrogen bonds with Asp-164, and Gln-269, and hydrophobic interactions with Asp-164, Pro-248, Tyr-264, and Gln-269. In Maestro, compound 5183914 was shown to have: π-π stacking with Tyr-268, and a hydrogen bond with Gln-269, in common with the reference ligand (Figure 7). When the results from PLIP and Maestro were combined (Supporting Information, Figure S4a–d), compound 121589399 was found to have six interactions in common with GRL-0617, whilst compounds 5384279 and 5183914 were found to have five and four interactions in common with the reference, respectively.

#### 2.3.2. Round 3: Docking the Analogues of the Best Hit from Round 2 

**Figure 8:**
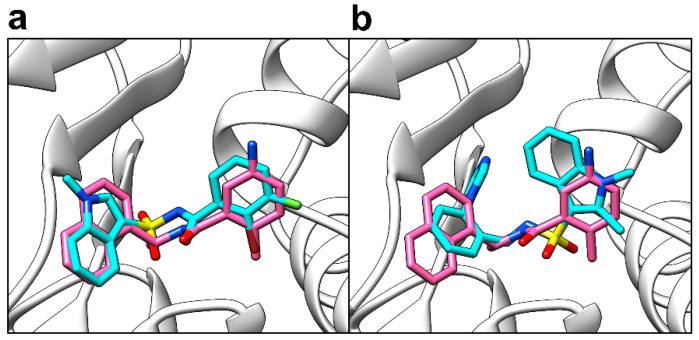
Poses of analogues from blind docking against PL^pro^ in AutoDock Vina. (**a**) Compound 121558793’s pose (cyan sticks) relative to the reference ligand GRL-0617’s co-crystallised pose (pink sticks); (**b**) Compound 132344896’s pose (cyan sticks) relative to the reference ligand GRL-0617’s co-crystallised (pink sticks). The protein is shown as white ribbons.

Of the two best scoring hits which had the most interactions in common with GRL-0617 (PubChem CIDs 121589399 and 5384279), analogues could only be found for PubChem CID 121589399. 27 commercially available analogues (Supporting Information, Figure S5; Supporting Information, Table S3) were obtained with a Tanimoto 2D similarity score of 0.7 and above using the MolPort SMILES and SMARTS search tool (www.molport.com). After docking these analogues, two hits were found with their mean predicted binding affinities closer to that of the reference ligand (Supporting Information, Table S4), with scores better (more negative) than −9.00. Compounds with PubChem CID 121558793 and PubChem CID 132344896 obtained ∆G values of −9.40 ± 0.00 kcal/mol (*n* = 5) and −9.26 ± 0.05 kcal/mol (*n* = 5), respectively, with poses in the same site as GRL-0617 (Figure 8) in five independent runs.

**Figure 9:**
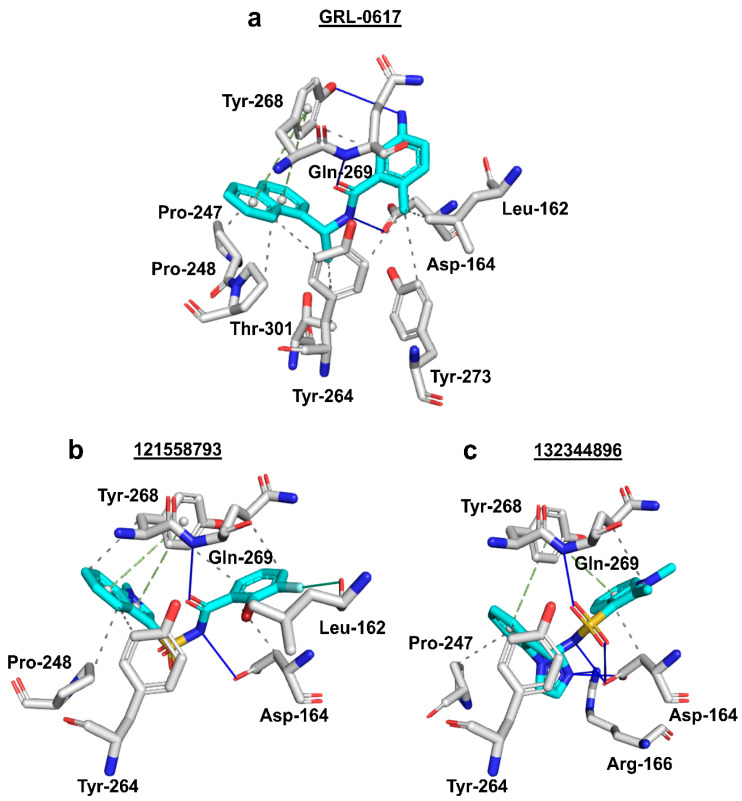
Three-dimensional protein-ligand interactions predicted by PLIP for two best scoring analogues of compound 121589399. (**a**) Interactions of reference ligand GRL-0617 (cyan sticks) with PL^pro^ residues (white/silver sticks); (**b**) Interactions of analogue compound 121558793 (cyan sticks) with PL^pro^ residues (white/silver sticks); (**c**) Interactions of analogue compound 132344896 (cyan sticks) with PL^pro^ residues (white/silver sticks). 3-letter codes and sequence numbers for amino acids are given. Hydrogen bonds are shown as blue lines. π-π stacking interactions are shown as green dashes. Hydrophobic interactions are shown as grey dots. The PubChem CID is shown above each compound.

Using PLIP, Compound 121558793 was found to have the following interactions in common with GRL-0617: π-π stacking with Tyr-268, hydrogen bonds with Asp-164 and Gln-269, and hydrophobic interactions with Asp-164, Pro-248, Tyr-264, Tyr-268, and Gln-269 (Figure 9a,b). Whilst compound 132344896 was found to have the following interactions in common with GRL-0617: π-π stacking with Tyr-268, hydrogen bonds with Asp-164 and Gln-269, and hydrophobic interactions with Asp-164, Pro-247, Tyr-264, and Gln-269 (Figure 9a,c). 

**Figure 10:**
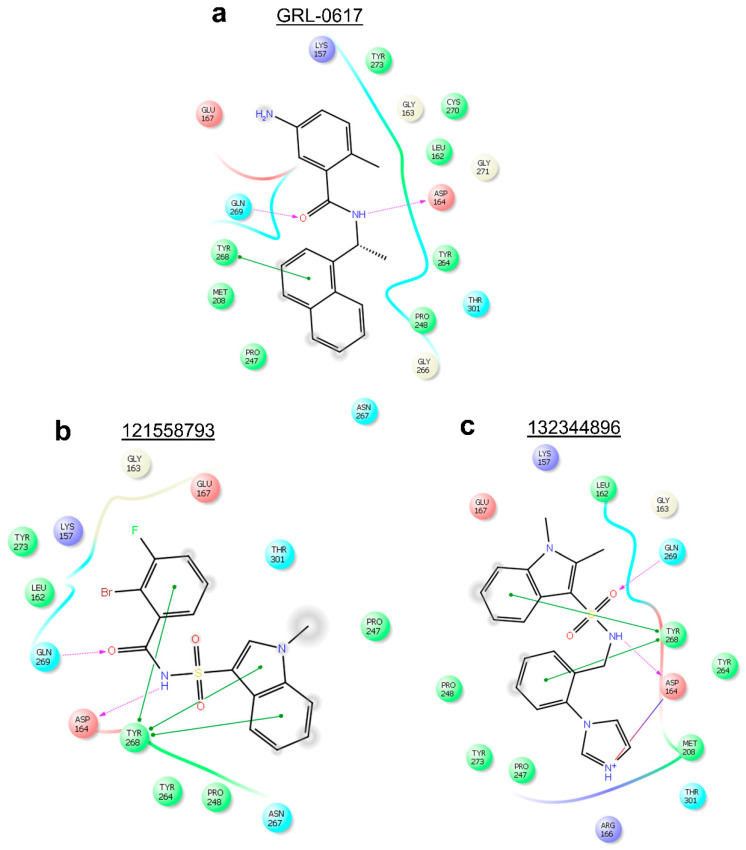
Two-dimensional protein-ligand interactions predicted for two best scoring analogues of compound 121589399 by Maestro. (**a**) Reference ligand GRL-0617’s interactions with PL^pro^ residues; (**b**) Compound 121558793’s interactions with PL^pro^ residues; (**c**) Compound 132344896’s interactions with PL^pro^ residues; π-π stacking interactions are represented by green lines. π-cation interactions are represented by red lines. Hydrogen bonds are represented by purple dashed arrows. Hydrophobic residues are shown as green circles. Positively charged residues are shown as purple circles. Negatively charged residues are shown as red circles. Polar residues are shown as cyan circles. Glycines are shown as light-yellow circles. A salt-bridge is shown as a multi-color (purple/red fusion) line. The PubChem CID is shown above each compound.

Additionally, analysis of protein-ligand interactions in Maestro showed that compounds 121558793 and 132344896 had all interactions in common with GRL-0617 (Figure 10). When the PLIP and Maestro results were combined (Supporting Information, Figure S4a,e,f), compounds 121558793 and 132344896 were found to have six and five interactions in common with the reference ligand, respectively.

#### 2.3.3. Refinement Using Focused Docking

**Figure 11:**
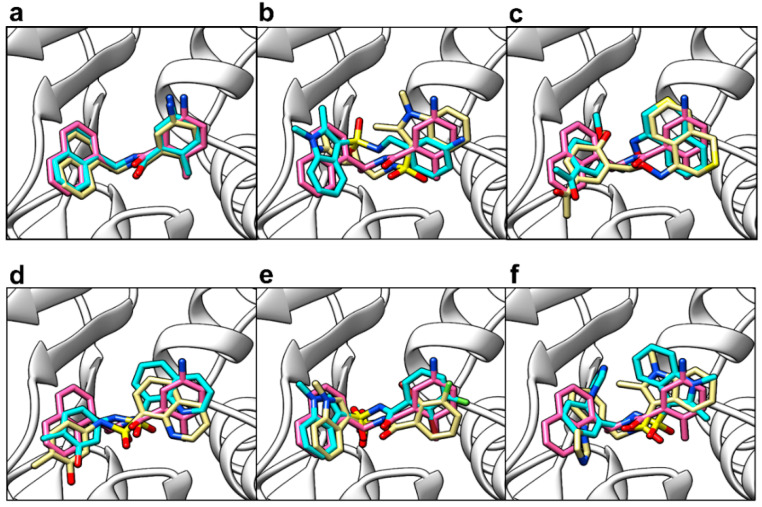
Comparison of poses from blind docking in Vina and focused docking (pose refinement) in AutoDock 4.2. The protein (PDB ID: 4JRN) is shown as white ribbons. (**a**) Reference ligand GRL-0617 docked pose from Vina (cyan sticks) and AutoDock 4.2 (gold sticks), relative to the co-crystallised pose (pink sticks); (**b**) Compound 121589399 docked pose from Vina (cyan sticks) and AutoDock 4.2 (gold sticks), relative to the co-crystallised pose of GRL-0617 (pink sticks); (**c**) Compound 5384279 docked pose from Vina (cyan sticks) and AutoDock 4.2 (gold sticks), relative to the co-crystallised pose of GRL-0617 (pink sticks); (**d**) Compound 5183914 docked pose from Vina (cyan sticks) and AutoDock 4.2 (gold sticks), relative to the co-crystallised pose of GRL-0617 (pink sticks); (**e**) Compound 121558793 docked pose from Vina (cyan sticks) and AutoDock 4.2 (gold sticks), relative to the co-crystallised pose of GRL-0617 (pink sticks); (**f**) Compound 132344896 docked pose from Vina (cyan sticks) and AutoDock 4.2 (gold sticks), relative to the co-crystallised pose of GRL-0617 (pink sticks).

Five hits entered focused docking in AutoDock 4.2 (Scripps Research, San Diego, CA, USA) [72,73], which were PubChem CID 121589399, PubChem CID 5384279, PubChem CID 5183914, PubChem CID 121558793, and PubChem CID 132344896. When GRL-0617’s docked pose obtained from AutoDock 4.2 was superimposed against its co-crystallised pose (Figure 11a), an RMSD of 0.672 Å was obtained (within the ≤2 acceptable range for validation of self-docking). Once again, the score and pose were highly reproducible in five independent docking runs (Supporting Information, Table S5). The mean predicted binding affinity calculated for GRL-0617 was −9.43 ± 0.05 (*n* = 5). Compound 121558793 (Figure 11e) was predicted by AutoDock 4.2 to have the best predicted binding affinity out of the five hits in focused docking (−9.17 ± 0.05 (*n* = 5)). The control and all five selected compounds obtained predicted binding affinities in the same rank as before (GRL-0617 < 121558793 < 132344896 < 121589399 < 5384279 < 5183914), in consensus with the blind docking results from AutoDock Vina. However, poses were not identical between the two programs. Where an alternative pose was suggested by AutoDock 4.2, it was usually a switch in orientation (e.g., Figure 11b), such that the compound’s ring group positions were flipped.

### 2.4. MM-GBSA Binding Energy Calculations

Selected docked compounds from the AutoDock 4.2-based focused docking were subjected to re-scoring through the Molecular Mechanics/Generalized Born Surface Area (MM-GBSA) method [68] implemented in Prime 3.0 (Schrödinger, L.L.C., New York, NY, USA) [74,75,76]. As shown in Table 1, the reference, GRL-0617, obtained the best (most negative) score of −61.9 ± 0.92 kcal/mol (*n* = 5). The molecule with the best score derived through this method was 121558793 (−57.5 ± 0.58 kcal/mol; *n* = 5), in consensus with the AutoDock Vina (blind docking) and AutoDock 4.2 (focused docking) results, which also ranked this molecule as the top in the set. In further agreement with the AutoDock tools, compound 132344896 obtained the second-best score of −52.01 ± 2.4 (*n* = 5).

The remaining three compounds obtained scores in the following order: 5384279 (−50.7 ± 1.6; *n* = 5), 121589399 (−40.3 ± 2.1; *n* = 5), and 5183914 (38.5 ± 2.4; *n* = 5). In contrast, the AutoDock tools had ranked compound 121589399 as higher than compound 5384279, however compound 5183914 had obtained the least desirable score in both the docking and MM-GBSA re-scoring.

### 2.5. Round 4: Scaffolds’ Drug-Likeness

The chemical structures of the five hits mentioned above were analysed to determine drug-likeness (Table 2), using the SwissADME tool (Swiss Institute of Bioinformatics, Quartier Sorge, Lausanne, Switzerland) [77], (www.swissadme.ch). The Lipinski (Pfizer, New York, NY, USA), Ghose (Amgen, Thousand Oaks, CA, USA), Veber (GlaxoSmithKline (GSK), Hong Kong), Egan (Pharmacia, Stockholm, Sweden), and Muegge (Bayer, Leverkusen, Germany) filters were used (Supporting Information, Table S6). Each of the five compounds complied with all the rules of drug-likeness constituted in each filter. Water solubility was predicted using the same tool and three compounds were predicted to be soluble (PubChem CIDs 121589399, 5183914, and 132344896), whilst two were predicted to be moderately soluble (PubChem CIDs 5384279 and 121558793). Four compounds all obtained an iLOGP > 2, reflecting good lipophilicity (121589399: 2.17, 132344896: 2.42, 5384279: 3.32, 121558793: 2.41), similar to GRL-0617’s iLOGP value of 2.69. However, compound 5183914 obtained an iLOGP of 1.54, reflecting poor lipophilicity. At this final stage, compound 5183914 was eliminated from the work as a promising scaffold, given it is unlikely to penetrate the plasma membrane of cells to reach the desired target protein in vitro. Lastly, medicinal chemistry analysis in the SwissADME tool revealed that none of the four remaining scaffolds were Pan Assay Interference Structures (PAINS) [78,79]. The compounds were also analysed for toxic chemical moieties (‘Brenk’ alerts) [80]. Compounds 121558793 and 132344896 contained no toxic groups. However, like GRL-0617, compound 121589399 contained an aniline group. Meanwhile, compound 5384279 was found to have two Brenk alerts: an imine group, and an oxygen-nitrogen single bond.

### 2.6. Predicted Off-Target Interactions

The similarity ensemble approach (SEA) was used to predict potential off-target interactions for the scaffolds by entering each compound’s SMILES into the SEA Search Server by Shoichet Lab (www.sea.bkslab.org). Only human proteins were considered potential targets. The server highlighted targets that had obtained *p*-values with an exponent of −16 (e-16), or more negative, as significant. GRL-0617 itself was predicted to interact with 14 human protein targets using the specified score threshold, including the potassium voltage-gated channel subfamily B member 2, extracellular calcium-sensing receptor, and glycogen phosphorylase (liver form). It was also predicted to interact with the prostaglandin E2 receptor EP4 subtype, ribonucleotide-diphosphate reductase (large subunit), ADP-ribosyl cyclase, ATP-dependent RNA helicase, cathepsin G, and peroxisome proliferator-activated receptor-gamma. Compound 121589399 was not predicted to interact with any human protein targets using the score threshold. Compound 5384279 was predicted to interact with one human protein target, the thyrotropin receptor. Compound 121558793 was predicted to interact with three human protein targets: RAS guanyl-releasing protein 1, prostaglandin E2 receptor EP3 subtype, and the prostaglandin F2-alpha receptor. Compound 132344896 was not predicted to interact with any human proteins using the score threshold. All the selected scaffolds were predicted to have fewer (or no) off-target interactions, compared to GRL-0617.

### 2.7. Scaffold Novelty and Scale of the Study

To evaluate scaffold novelty, we used our four hits to screen 1474 molecules, and 8904 molecules, annotated with the keyword ‘SARS-CoV-2’ in PubChem (www.pubchem.ncbi.nlm.nih.gov), and the ChemBL database [81] (www.ebi.ac.uk/chembl), respectively, using DataWarrior’s, version 5.2.1 (www.openmolecules.org) [82] default fingerprint descriptor FragFp, but found no hits. Likewise, we found no similar hits when we analysed the structures submitted to the COVID Moonshot Initiative [83] (www.covid.postera.ai/covid), and IUPHAR/BPS Guide to Pharmacology [36,37] (www.guidetopharmacology.org). Additionally, the compounds’ PubChem Bioassay profiles were also checked. The scaffolds were found to be novel in all cases, with none previously tested against the PL^pro^, or submitted for testing. Three of the molecules have no PubChem Bioassay data, whilst compound 5384279 has one PubChem bioassay record (Bioassay ID:1159607), where the molecule was found inactive against a non-viral target protein. Moreover, using keyword searches in PubMed (www.pubmed.ncbi.nlm.nih.gov), Web of Science (www.webofknowledge.com), Scopus (www.scopus.com), and Google Scholar (www.scholar.google.com), papers were collected which involved virtual screening of novel molecules (but not approved drugs) against PL^pro^. From the collection of papers we could find and analyse, our virtual screen was found to be the largest ligand-based screen against GRL-0617. Additionally, we did not find any of our scaffolds previously mentioned in any papers.

## 3. Discussion

In this work, we present the largest virtual screen against the PL^pro^ non-covalent inhibitor GRL-0617 which, in combination with multiple rounds of docking, resulted in the identification of four novel, lipophilic, and commercially available, scaffolds for in vitro validation against PL^pro^ and viral replication. The scale of exploration meant we could search unchartered and diverse regions of chemical space to find the most similar scaffolds to GRL-0617. Our integrative 4-step approach consisted of a ligand-based screen where shape, groups, and electrostatic fields of compounds were analysed, and a structure-based modelling approach that explored the compounds’ predicted binding affinity, location, and poses against PL^pro^, as well as protein-ligand interactions. Analogues were found for the best hit from blind docking, which led to two additional scaffolds with better predicted binding affinities. The docking results were further refined using MM-GBSA calculations. Finally, leads were all evaluated using various pharma rules of drug-likeness to ensure only the most promising scaffolds are presented for further testing. Predicted off-target interactions were also explored. Importantly, we intentionally focused on libraries of commercially available small-molecules to ensure that research groups will be able to purchase, and validate, our hits against the virus in vitro. 

Although molecules obtained from virtual screens have previously been deposited in the literature for prospective testing against PL^pro^ [84,85,86,87,88], these were mainly docking studies, and we could not find any paper where a ligand-based virtual screen of such scale had been performed against GRL-0617 before docking. Additionally, and in contrast to our work, the docking had commonly been performed against the PL^pro^ catalytic site and not the GRL-0617 binding site, which is distinctly different. Furthermore, all our scaffolds are novel and have not previously been tested against the PL^pro^, or presented for testing, when checked against several databases. Aside from GRL-0617, which has been reported by the IUPHAR/BPS Guide to Pharmacology as the only PL^pro^ inhibitor to date (with a peer-reviewed article), and the only small-molecule inhibitor which has been co-crystallised with PL^pro^ [40], other inhibitory scaffolds against PL^pro^ with experimental validation do exist [38]. The dissemination of such scaffolds is critical to drug discovery and development, and the higher the number of structure-activity relationships delineated, and functional data available, we can begin to establish the key pharmacophore features required for a specific and potent antiviral inhibitor against PL^pro^. The four scaffolds we present in this work may enrich this small collection, and further inform drug discovery efforts. Importantly, the scaffolds were predicted to have none or far fewer off-target interactions than GRL-0617, which is a desired feature in the drug discovery, process, where the availability of selective scaffolds may facilitate the development of safer medicines with fewer side effects [89,90]. Moreover, compounds 121558793 and 132344896 had no toxic chemical moieties.

The use of GRL-0617 as a reference in the virtual screens facilitated the initial identification of 24 distinct scaffolds. Scaffold hopping has consistently been employed as an important tool in drug discovery and has yielded many active ligands thus far [91,92,93]. The availability of more than one scaffold is an advantageous starting point in any drug development process, given that attrition rates are high due to either lack of potency or poor pharmacokinetic profiles. In the ligand-based work, compound 121589399 had notably obtained the best Shape Tanimoto score, and a field score of above 0.8, and as well as the most favourable predicted free energy of binding in the initial blind docking. ROCS and Forge have previously been demonstrated to yield bioactive ligands [67,94]. Before blind docking, we first tested several programs to find out which would reproduce the co-crystallised GRL-0617 pose with the lowest RMSD between docked and co-crystallised pose, in which AutoDock Vina outperformed the other programs.

In our docking studies, we used the two AutoDock tools created by the Scripps Research Institute (San Diego, CA, USA): firstly, AutoDock Vina for blind docking, and subsequently, AutoDock 4.2 for focused docking. Our aim in the focused docking round was to establish whether the scores and poses from AutoDock Vina could be reproduced in a related program with a different docking algorithm. In the analogue-based work, both programs had predicted compound 121558793 to have the most favourable predicted binding affinity against the GRL-0617 binding site. Compounds that obtain promising scores in different programs are often regarded as promising hits in computational work [95]. Additionally, the score rank of compounds was the same in both programs (GRL-0617 < 121558793 < 132344896 < 121589399 < 5384279 < 5183914). However, AutoDock 4.2 suggested alternative poses for some of the compounds, than that obtained in AutoDock Vina. Interestingly, it has been reported by numerous studies that AutoDock Vina outperforms AutoDock 4.2 in pose prediction [69,96,97,98,99,100], which is why we rely on our presented AutoDock Vina poses and their respective protein-ligand interactions. Furthermore, a correlation has been suggested between Vina scores and in vitro pIC_50_ values [100]. Vina has previously yielded multiple active ligands in vitro [101,102,103,104]. 

Vina’s algorithm is based on a hybrid scoring function (empirical and knowledge-based) [69], whereas AutoDock 4.2’s scoring algorithm is based on the Assisted Model Building with Energy Refinement (AMBER) force field [72]. Physics-based scoring functions, such as that employed by AutoDock 4.2, have previously been regarded as less accurate in pose prediction than knowledge-based scoring functions [100]. However, in another study AutoDock 4.2 has been reported as more accurate in predicting free energy of binding (scores), than AutoDock Vina [98]. In either case, our use of AutoDock 4.2 for focused docking was useful for establishing patterns in compound scores between the two programs, and since the compounds’ rank in both programs was identical, deciding on which program was the more accurate determiner of scores did not seem to matter much in this case. Both programs had predicted the control ligand to have the best predicted binding affinity, which demonstrated the accuracy in predicting power of the two algorithms. Furthermore, we ran five independent docking runs for each compound, due to the stochastic nature of the programs. The programs could repeatedly reproduce the co-crystallised pose of GRL-0617, further reflecting their sampling power, and emphasising their reliability to dock the test scaffolds. Additionally, when we performed Molecular Mechanics/Generalised Born Surface Area (MM-GBSA) calculations in Prime, the top-scoring molecules were 121558793 and 132344896, in agreement with the focused and blind docking results from the AutoDock tools. Hence, re-scoring the docked compounds’ free energy of binding using the MM-GBSA method allowed us to further refine and validate the docking results. Previously, such re-scoring with MM-GBSA has provided good correlation with experimentally-obtained binding data [105,106,107,108,109].

Moreover, we used a two-dimensional and three-dimensional approach (and a combination of the two) when analysing protein-ligand interactions of poses obtained from the docking programs, which allowed us to perform an in-depth analysis of predicted residues involved in ligand binding. Our approach was also highly stringent, as we considered all of the control ligand’s interactions as criteria to compare the test compounds’ interactions against, since it is not fully known which, or how many of the predicted residues, play a central role in GRL-0617 binding. In general, although the protein-ligand interactions predicted by PLIP (3D) and Maestro (2D) cannot be directly compared due to their different prediction algorithms, and number of predicted interactions, using both programs allowed us to build a multi-layered and comprehensive perspective of protein-ligand interactions.

Our analogue-based approach, which was based on compound 121589399, facilitated the discovery of two hits with closer predicted binding affinities to the control ligand, which reflected the practicality and usefulness of such an approach. Additionally, analogues of our hits may be further explored in future work to find other scaffolds with favourable predicted binding affinities in the search for more potent inhibitors of PL^pro^. As these scaffolds were found using the GRL-0617 structural template, they may also facilitate establishing SAR around the GRL-0617 scaffold. This may be useful in the design of more potent inhibitors. Importantly, aside from potency, collections of our and other scaffolds may be useful in structural optimization to achieve desirable pharmacokinetic profiles [110]. This is because, for any drug discovery initiative, it is advisable to start with several distinct scaffolds as hits or leads, as not all scaffolds will necessarily survive various rigorous steps during the pre-clinical stage.

Thus far, there is no approved drug against the SARS coronaviruses. The growing worldwide mortality rate resulting from the COVID-19 crisis demands an urgent need for an effective inhibitor. An approved drug against SARS-CoV-2 may likely be repurposed against another novel coronavirus. Hence, the availability of multiple inhibitory scaffolds may be of paramount importance in our toolkit to develop safe medicines in the fight against future coronavirus pandemics. This is because an available drug may dramatically lower mortality rates and help to contain epidemics at the source to prevent worldwide transmission. Although an approved vaccine is also of critical importance in preventing morbidity, it may also be considered that a vaccine may not retain its efficacy against a mutated form of the virus [111,112,113]. However, this problem may still be overcome with an approved drug against either M^pro^ or PL^pro^ (or a ‘gold-standard’ cocktail drug with a combination of molecules against both). The four novel scaffolds we present in this work may be employed for experimental validation in the pursuit of novel inhibitory scaffolds against the SARS-CoV-2 PL^pro^. 

## 4. Materials and Methods 

### 4.1. Chemical Libraries

Three chemical libraries containing novel screening compounds were obtained for ligand-based virtual screening-the ChemDiv Diversity^®^ library (52,000 small-molecules) (www.chemdiv.com), the MayBridge Hit Locator^®^ library (53,000 small-molecules) (www.maybridge.com), and the Enamine Hit Locator^®^ library (234,240 small-molecules) (www.enamine.net).

### 4.2. Ligand-Structure Preparation

The three-dimensional structures of molecules contained in the screening libraries, as well as the co-crystallised non-covalent inhibitor, GRL-0617, which had been obtained from PubChem (www.pubchem.ncbi.nlm.nih.gov), were energy-minimised in the Molecular Operating Environment (MOE) [114], version 2019.01 (Chemical Computing Group, Montreal, QC, Canada).

### 4.3. Ligand-Based Virtual Screening

ROCS [63,64], version 3.4.1.0 (OpenEye Scientific Software, Santa Fe, NM, USA), was employed for virtual screening. The three-dimensional structure of GRL-0617 was used as the query molecule. The three libraries were screened for hits with similar 3D shape/structural and chemical similarity to GRL-0617. The Shape Tanimoto score denoted structural similarity (maximum obtainable score: 1), and the Color Tanimoto score (maximum obtainable score: 1) denoted chemical group similarity. A ROCS Report was created using the OpenEye command line. Subsequently, the highest-scoring hits were aligned to the query molecule GRL-0617 in Forge [65,66,67], version 10.4.2 (Cresset, Litlington, Cambridgeshire, UK), and electrostatic field similarity was manually inspected to select a sub-set of molecules for molecular docking. The molecules’ field scores were noted.

### 4.4. Protein-Structure Preparation

The three-dimensional protein structure of the SARS-CoV-2 PL^pro^ was obtained from the PDB (www.rcsb.org) (PDB ID: 7JRN). The protein was prepared for molecular docking using ICM-Pro [115], version 3.8 (Molsoft, L.L.C., San Diego, CA, USA). These preparations included the removal of water molecules and ligands and the addition of hydrogen groups.

### 4.5. Molecular Docking

#### 4.5.1. Blind Docking

Validation blind docking was first performed using different docking software to find the program which could best reproduce the GRL-0617 co-crystallised pose. AutoDock Vina, version 1.1.2 (Scripps Research, San Diego, CA, USA; www.scripps.edu) [69] (also referred to as just ‘Vina’), AutoDock 4.2, version 4.2.6 (also referred to as ‘AD 4.2’, or ‘AutoDock 4’) [72,73], SwissDock webserver (www.swissdock.ch) [116], and the Genetic Optimisation for Ligand Docking (GOLD) suite, version 5.8.0 [117] (The Cambridge Crystallographic Data Centre, Cambridge, Cambridgeshire, UK), were tested. An exhaustiveness value of 24 was used in AutoDock Vina, with the AutoGrid maximised to encapsulate the entire structure of the protein. In AutoDock 4.2, the AutoGrid was also maximised, and the Lamarckian genetic algorithm was opted for. The zinc ion was removed before docking in AutoDock 4.2. AutoDock Vina and AutoDock 4.2 were both run in the PyRx [118] interface, version 0.8 (www.sourceforge.net).Compounds were converted to ‘AutoDock Ligand’ using OpenBabel [119], version 2.4.0 (also in PyRx), and the protein structure was imported and converted to ‘AutoDock Macromolecule’ before docking. ‘Pseudo’ blind docking was performed in GOLD. The binding site was defined as the center mass of the bound GRL-01617 (X, Y, Z, coordinates: 10.875, Y: −11.327, 31.594, respectively). Atoms within a 10 Å radius of this location were used as the search space for blind docking. For each program, the highest-ranked pose with the best (most negative) predicted free energy of binding, Gibbs free energy (ΔG) in(kcal/mol), or ChemPLP score (GOLD), was superimposed against the GRL-0617 co-crystallised pose, to calculate an RMSD value in DockRMSD, version 1.1 [120] (Zhang Lab, University of Michigan; www.zhanglab.ccmb.med.umich.edu/DockRMSD). The AutoDock tools measure predicted free energy of binding (ΔG), but scores are commonly reported as predicted binding affinity. Thus, the terms ‘ΔG’ and ‘binding affinity’ are used interchangeably throughout the text. Where mean ± SEM is shown, five independent docking runs were performed to calculate these values. All presented docking poses are that of the highest-ranked pose. The highest-ranking pose for each compound was superimposed against the GRL-0617 co-crystallised pose, in UCSF Chimera [121], version 1.14 (University of California, San Francisco, CA, USA).

#### 4.5.2. Refinement Using Focused Docking

The best molecules from blind docking were further analysed using focused docking, which was performed using AutoDock 4.2 [72]. The molecule poses from blind docking were converted to ‘AutoDock Ligand’ using OpenBabel, version 3.3.0, implemented in the PyRx interface. Before importing the protein structure, its zinc ion was removed. The AutoGrid was focused-in on the GRL-0617 binding site. The Lamarckian genetic algorithm was opted for. Validation of the docking protocol was performed using GRL-0617. The highest-ranking pose for each compound was superimposed against its Vina blind docking pose, and the GRL-0617 co-crystallised pose, in UCSF Chimera [121].

### 4.6. MM-GBSA Binding Energy Calculations

Selected docked compounds were subjected to re-scoring through the Molecular Mechanics/Generalized Born Surface Area (MM-GBSA) method implemented in Prime, version 3.0 (Schrödinger, L.L.C., New York, NY, USA) [74,75,76] as per published protocol [68]. The relative free energy of binding (ΔG, kcal/mol) was calculated for each molecule as an average of five independent runs of the Prime MM-GBSA protocol. 

### 4.7. Analogue Search

The MolPort SMILES and SMARTS search tool (www.molport.com/shop/find-chemicals-by-smiles) was used to find analogues. A 2D Tanimoto cutoff of 0.7 was used. The analogues were energy-minimised and docked, as described above.

### 4.8. Analysis of Protein-Ligand Interactions

PLIP (BIOTEC, Tatzberg, Dresden; www.projects.biotec.tu-dresden.de/plip-web/plip) [70] was used to analyse protein-ligand interactions in 3D. Additionally, 2D protein-ligand interaction diagrams were created using the Maestro suite (Schrödinger, L.L.C., New York, NY, USA) [71].

### 4.9. Molecular Visualization 

PyMOL, version 2.4 (Schrödinger, L.L.C., New York, NY, USA) and UCSF Chimera [121] were used to visualise poses against the protein binding pocket. Open Babel and Molegro Molecular Viewer (MMV) [122], version 7.0 (Molexus IVS, Odder, Denmark)) were used for processing files from docking. MarvinSketch, version 20.16 (ChemAxon Ltd., Budapest, Hungary) [123] was used to draw two-dimensional chemical structures.

### 4.10. Assessing Molecules’ Drug-Likeness

The molecules’ SMILES were entered into the SwissADME web server [77] (www.swissadme.ch) to determine drug-likeness using the Lipinski (Pfizer), Ghose (Amgen), Veber (GlaxoSmithKline), Egan (Pharmacia), and Muegge (Bayer) filters, with each having set rules for drug-likeness (Supporting Information, Table S6). The SwissADME tool was also used to predict the compounds’ water solubility using Log S (Ali) [124] and lipophilicity with calculated iLOGP [125,126] values, as well as their medicinal chemistry (PAINS analysis [78,79], and toxic chemical moieties (Brenk alerts) [80]).

### 4.11. Predicted Off-Target Interactions

The similarity ensemble approach (SEA) was used to predict potential off-target interactions for the scaffolds by entering each compound’s SMILES into the SEA Search Server by Shoichet Lab (www.sea.bkslab.org). Only human proteins were considered. The server highlighted targets that had obtained *p*-values with an exponent of −16 (e-16), or more negative, as significant.

### 4.12. Scaffold Novelty and Scale of the Study

To evaluate scaffold novelty, we used our five hits to screen 1474 molecules and 8904 molecules annotated (accessed on 14 February 2021) with the keyword ‘SARS-CoV-2’ in PubChem (www.pubchem.ncbi.nlm.nih.gov) and the ChemBL database [81] (www.ebi.ac.uk/chembl), respectively, using DataWarrior’s, version 5.2.1, (www.openmolecules.org) [82] default fingerprint descriptor FragFp. Likewise, the COVID Moonshot Initiative [83] (www.covid.postera.ai/covid/structures), IUPHAR/BPS Guide to Pharmacology [36,37] (www.guidetopharmacology.com), and PubChem Bioassay data, were searched to confirm scaffold novelty. 

To confirm whether this work contained the largest virtual screen against the SARS-CoV-2 PL^pro^, keyword searches were performed in PubMed (www.pubmed.ncbi.nlm.nih.gov), Web of Science (www.webofknowledge.com), Scopus (www.scopus.com), and Google Scholar (www.scholar.google.com) to find papers where virtual ligand-based screens had been conducted in the search for novel inhibitors against PL^pro^. Any hits presented in such papers were also visually compared to our hits to further check scaffold novelty.

## Figures and Tables

**Table 1:** The relative free energy of binding of selected molecules through MM-GBSA based re-scoring.

Molecule	MM-GBSA Re-Scoring (ΔG_bind_, kcal/mol) *
GRL-0617	−61.9 ± 0.92
5183914	−38.5 ± 2.4
121589399	−40.3 ± 2.1
5384279	−50.7 ± 1.6
121558793	−57.5 ± 0.58
132344896	−52.01 ± 2.4

* values represent mean ± SEM from *n* = 5 independent runs of MM-GBSA protocol implemented in Prime 3.0 (Schrödinger, L.L.C., New York, NY, USA) [76].

**Table 2:** Evaluation of scaffolds’ drug-likeness using SwissADME tool. The results show that the reference GRL-0617 and selected scaffolds (PubChem CIDs are shown) successfully passed five different filters with individual pharma rules of drug-likeness. Also shown are compounds’ solubility, lipophilicity, and 2D structures (drawn in MarvinSketch).

	GRL-0617	121589399	5384279	5183914	121558793	132344896
Lipinski (Pfizer)	✓	✓	✓	✓	✓	✓
Ghose (Amgen)	✓	✓	✓	✓	✓	✓
Veber (GSK)	✓	✓	✓	✓	✓	✓
Egan (Pharmacia)	✓	✓	✓	✓	✓	✓
Muegge (Bayer)	✓	✓	✓	✓	✓	✓
Water solubility (Log S (Ali))	Moderate (−4.89)	Soluble (−3.61 )	Moderate (−4.91)	Soluble (−3.22)	Moderate (−4.48)	Soluble (−3.88)
Lipophilicity (iLOGP)	Good 2.69	Good 2.17	Good 3.32	Poor 1.54	Good 2.41	Good 2.42
Structure	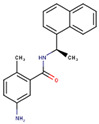	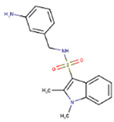	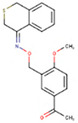	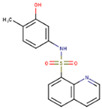	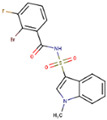	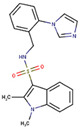

## Supplementary Materials

The following are available online. Figure S1: Two-dimensional structures of 24 hits obtained from ligand-based virtual screening; Table S1: Commercial availability of 24 hits obtained from ligand-based screens, Figure S2: ROCS result for 24 hits from ligand-based screening; Figure S3: Hits’ electrostatic field comparison to the reference (Forge results); Table S2: Vina results for docking control and 24 hits from ligand-based screening against PL^pro^; Figure S4: Venn diagrams showing the combination of PLIP and Maestro results for the reference and five selected hits; Figure S5: Two-dimensional structures of the analogues of compound 121589399; Table S3: Commercial availability of 27 analogues of compound 121589399; Table S4: Vina results for docking control and 27 analogues of compound 121589399; Table S5: Focused docking of the control and the five selected hits in AutoDock 4.2; Details of drug-likeness filters (rules) used in the SwissADME tool.

## Sample Availability

Samples of the compounds are not available from the authors.
