# Supplementary material for: Establishing an Analogue Based In Silico Pipeline in the Pursuit of Novel Inhibitory Scaffolds against the SARS Coronavirus 2 Papain-Like Protease
Source: Molecules. 2021 Feb 20;26(4):1134. doi: 10.3390/molecules26041134 (PMC7924369; doi:10.3390/molecules26041134)
Supplement: Supplementary file 1 [file molecules-26-01134-s001.pdf]

Establishing an Analogue-Based in Silico Pipeline in the Pursuit of Novel Inhibitory Scaffolds Against the SARS Coronavirus 2 Papain-Like Protease

Supplementary Material

Roxanna Hajbabaie, Matthew T. Harper, Taufiq Rahman*

Cambridge University, Department of Pharmacology

*Correspondence: mtur2@cam.ac.uk

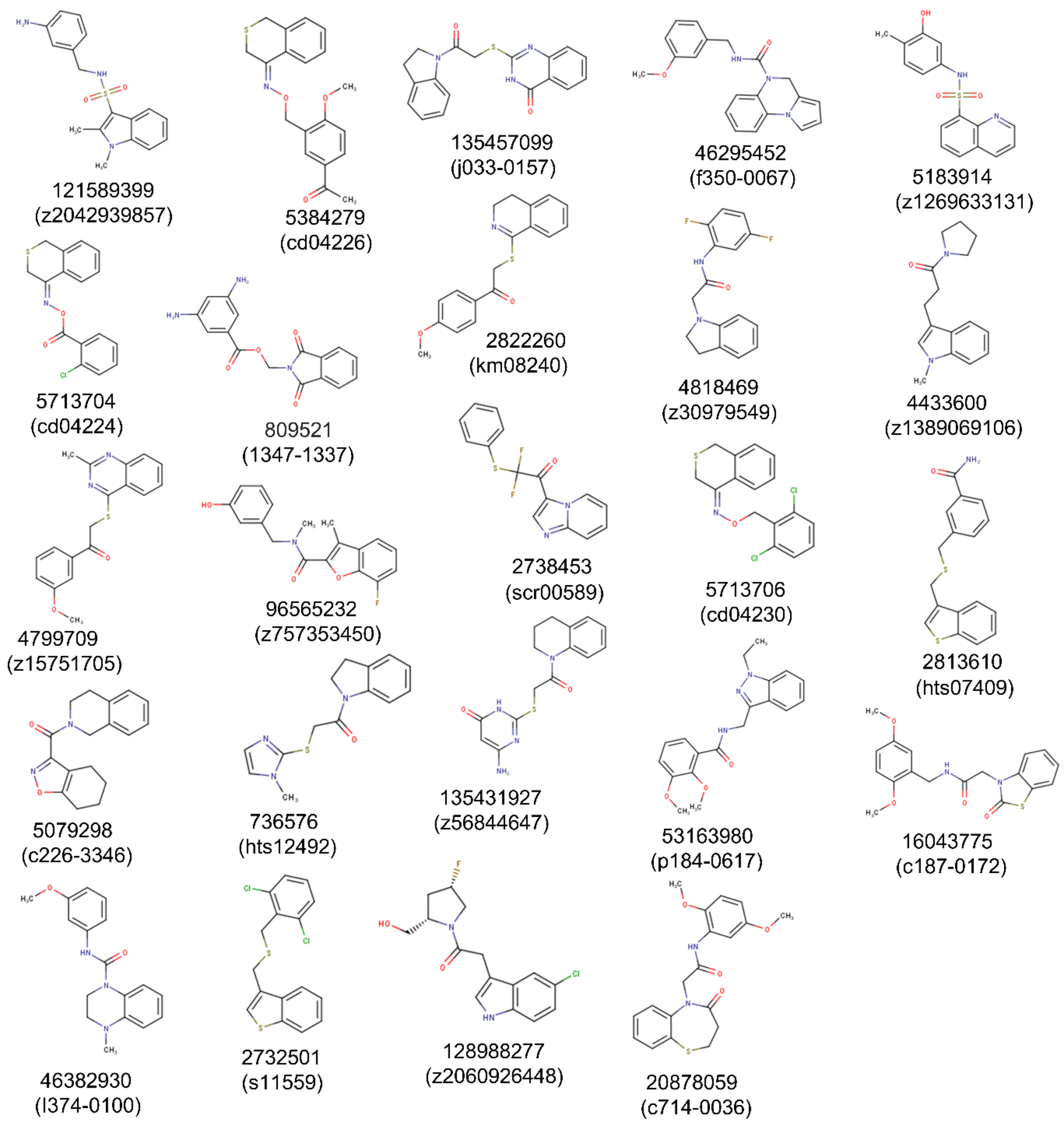

Figure S1: Two-dimensional structures of 24 hits obtained from ligand-based virtual screening. The structures were drawn using MarvinSketch. The PubChem CID of the compound is shown. The vendor's compound ID is shown in brackets.

Table S1: Commercial availability of 24 hits obtained from ligand-based screens.

PubChem CID	Vendor	Vendor ID	MolPort ID	SMILES
**121589399**	Enamine	z2042939857	MolPort-039-320-847	CC1=C(C2=CC=CC=C2N1C)S(=O)(=O)NCC3=CC(=CC=C3)N
**5384279**	MayBridge	cd04226	MolPort-001-806-103	CC(=O)C1=CC(=C(C=C1)OC)CO/N=C\2/CSCC3=CC=CC=C32
**135457099**	ChemDiv	j033-0157	MolPort-001-008-572	C1CN(C2=CC=CC=C21)C(=O)CSC3=NC4=CC=CC=C4C(=O)N3
**46295452**	ChemDiv	f350-0067	MolPort-007-795-945 (discontinued)	COC1=CC=CC(=C1)CNC(=O)N2CC3=CC=CN3C4=CC=CC=C42
**5183914**	Enamine	z1269633131	MolPort-023-167-269	CC1=C(C=C(C=C1)NS(=O)(=O)C2=CC=CC3=C2N=CC=C3)O
**5713704**	MayBridge	cd04224	MolPort-002-895-291	C1C2=CC=CC=C2/C(=N\OC(=O)C3=CC=CC=C3Cl)/CS1
**809521**	ChemDiv	1347-1337	MolPort-001-888-052	C1=CC=C2C(=C1)C(=O)N(C2=O)COC(=O)C3=CC(=CC(=C3)N)N
**2822260**	MayBridge	km08240	MolPort-001-809-107	COC1=CC=C(C=C1)C(=O)CSC2=NCCC3=CC=CC=C32.Br
**4818469**	Enamine	z30979549	MolPort-004-129-267	C1CN(C2=CC=CC=C21)CC(=O)NC3=C(C=CC(=C3)F)F
**4433600**	Enamine	z1389069106	MolPort-027-905-390	CN1C=C(C2=CC=CC=C21)CCC(=O)N3CCCC3
**4799709**	Enamine	z15751705	MolPort-004-101-729	CC1=NC2=CC=CC=C2C(=N1)SCC(=O)C3=CC(=CC=C3)OC
**96565232**	Enamine	z757353450	MolPort-038-952-115	CC1=C(OC2=C1C=CC=C2F)C(=O)N(C)CC3=CC(=CC=C3)O
**2738453**	MayBridge	scr00589	MolPort-002-919-534	C1=CC=C(C=C1)SC(C(=O)C2=CN=C3N2C=CC=C3)(F)F
**5713706**	Maybridge	cd04230	MolPort-002-895-294	C1C2=CC=CC=C2/C(=N\OCC3=C(C=CC=C3Cl)Cl)/CS1
**2813610**	MayBridge	hts07409	MolPort-001-808-409	C1=CC=C2C(=C1)C(=CS2)CSCC3=CC(=CC=C3)C(=O)N
**5079298**	ChemDiv	c226-3346	MolPort-007-603-904 (discontinued)	C1CCC2=C(C1)C(=NO2)C(=O)N3CCC4=CC=CC=C4C3

Table S1 continued.

PubChem CID	Vendor	Vendor ID	MolPort ID	SMILES
**736576**	MayBridge	hts12492	MolPort-001-842-262	CN1C=CN=C1SCC(=O)N2CCC3=CC=CC=C32
**135431927**	Enamine	z56844647	MolPort-002-952-534	C1CC2=CC=CC=C2N(C1)C(=O)CSC3=NC(=CC(=O)N3)N
**53163980**	ChemDiv	p184-0617	MolPort-010-922-186	CCN1C2=CC=CC=C2C(=N1)CNC(=O)C3=C(C(=CC=C3)OC)OC
**16043775**	ChemDiv	c187-0172	MolPort-007-594-123	COC1=CC(=C(C=C1)OC)CNC(=O)CN2C3=CC=CC=C3SC2=O
**46382930**	ChemDiv	L374-0100	MolPort-007-964-161	CN1CCN(C2=CC=CC=C21)C(=O)NC3=CC(=CC=C3)OC
**2732501**	MayBridge	s11559	MolPort-002-918-462	C1=CC=C2C(=C1)C(=CS2)CSCC3=C(C=CC=C3Cl)Cl
**128988277**	Enamine	z2060926448	MolPort-039-172-558	C1[C@@H](CN([C@@H]1CO)C(=O)CC2=CNC3=C2C=C(C=C3)Cl)F
**20878059**	ChemDiv	c714-0036	MolPort-007-661-724	COC1=CC(=C(C=C1)OC)NC(=O)CN2C(=O)CCSC3=CC=CC=C32

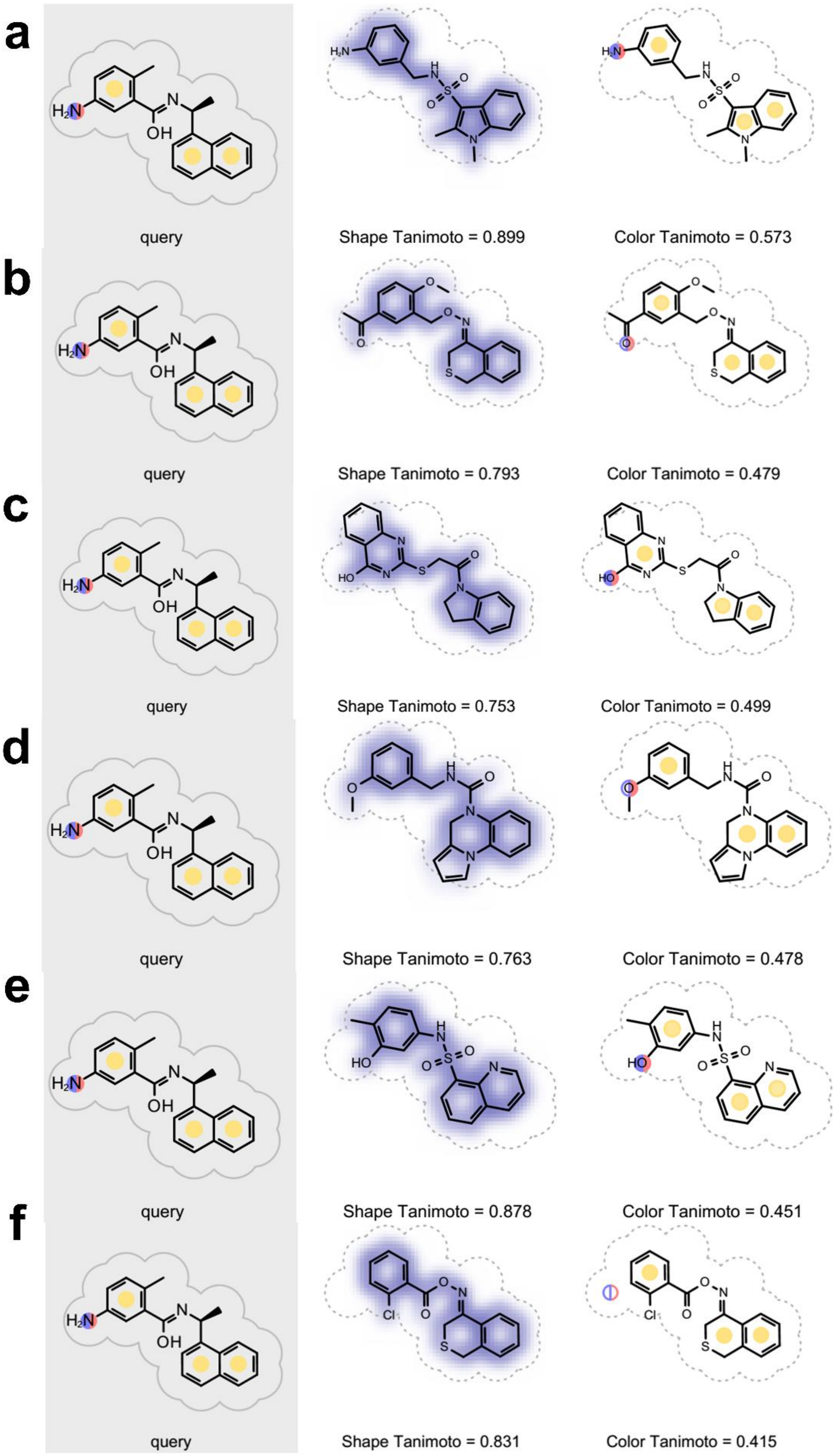

Figure S2: ROCS result for 24 hits from ligand-based screening. Query is GRL-0617**.** PubChem CIDs: **a)** 121589399, **b)** 5384279, **c)** 135457099, **d)** 46295452, **e)** 5183914, **f)** 5713704.

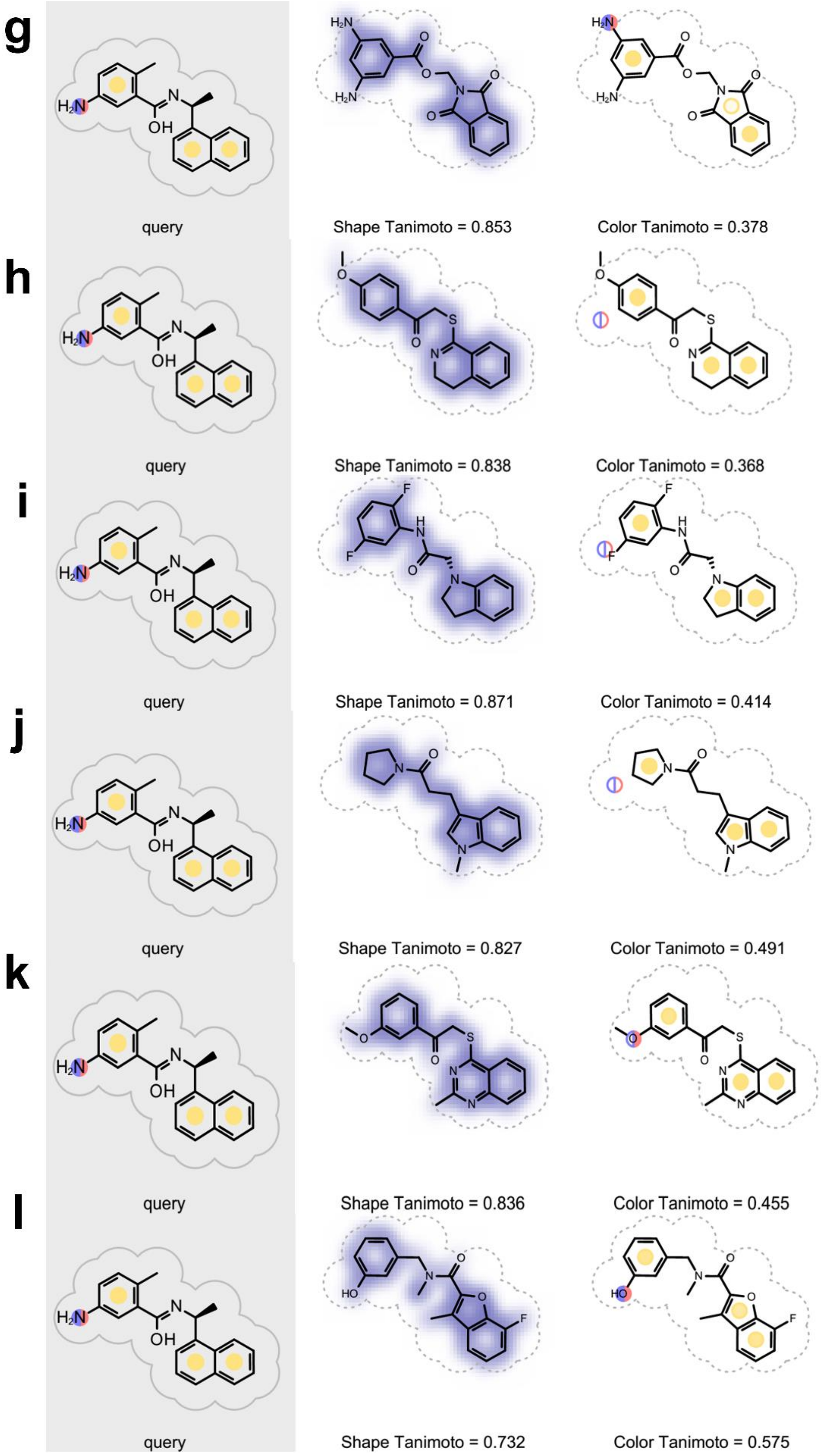

Figure S2 continued. PubChem CIDs: **g)** 809521, **h)** 2822260, **i)** 4818469, **j)** 4433600, **k)** 4799709, **l)** 96565232.

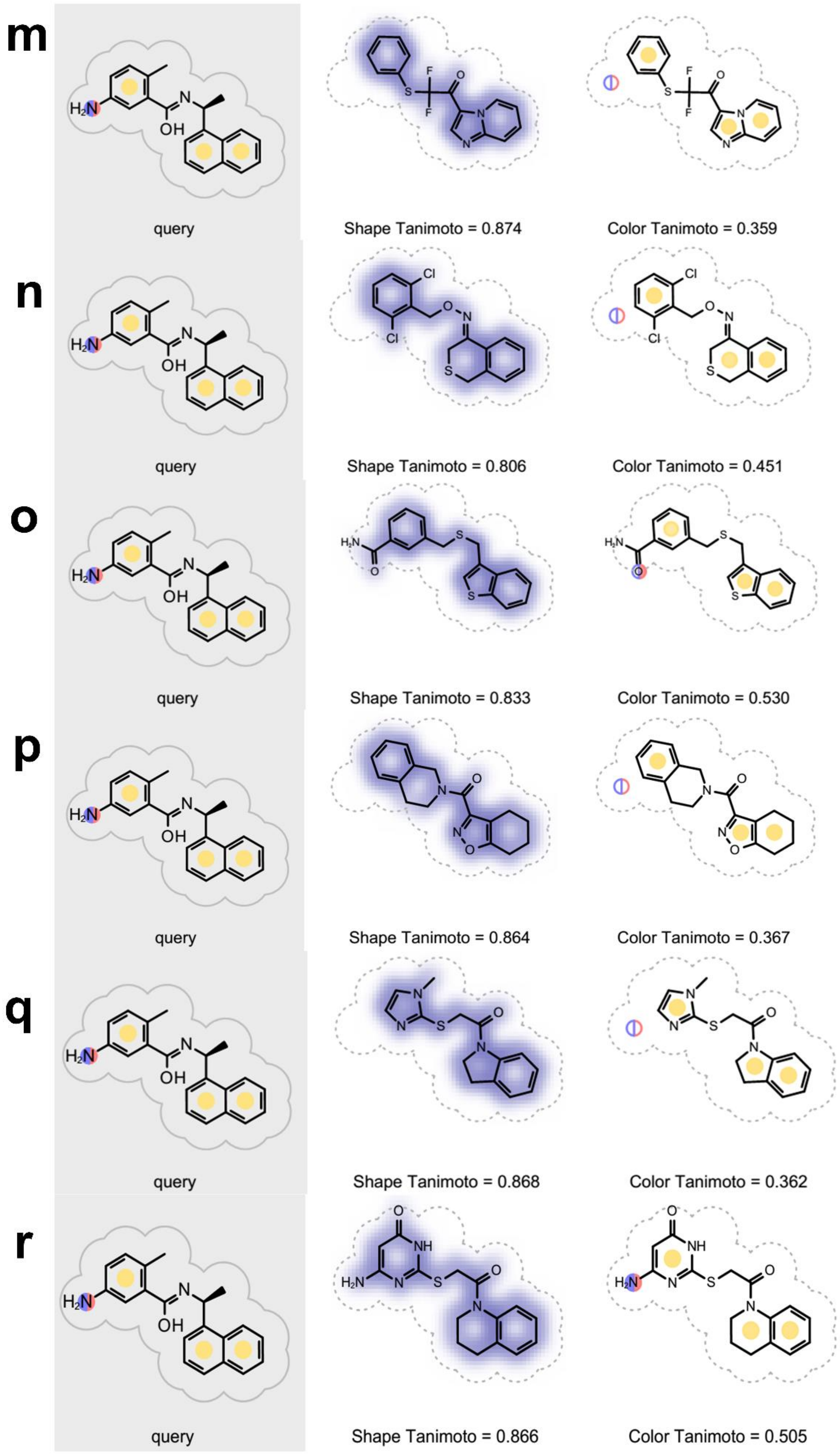

Figure S2 continued. PubChem CIDs: **m)** 2738453, **n)** 5713706, **o)** 2813610, **p)** 5079298, **q)** 736576, **r)** 135431927.

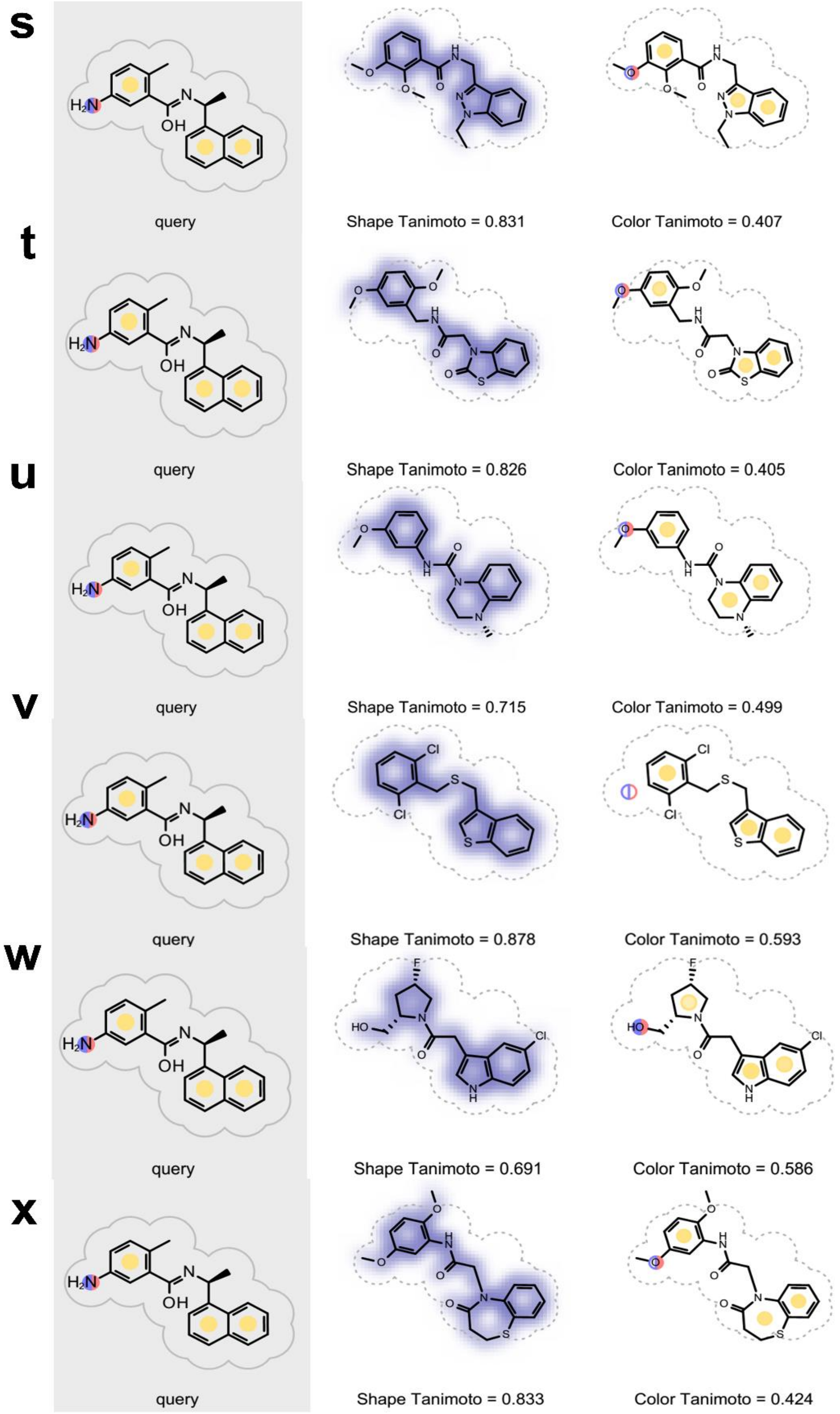

Figure S2 continued. PubChem CIDs**: s)** 53163980**, t)** 16043775, **u)** 46382930, **v)** 2732501, **w)** 128988277, **x)** 20878059

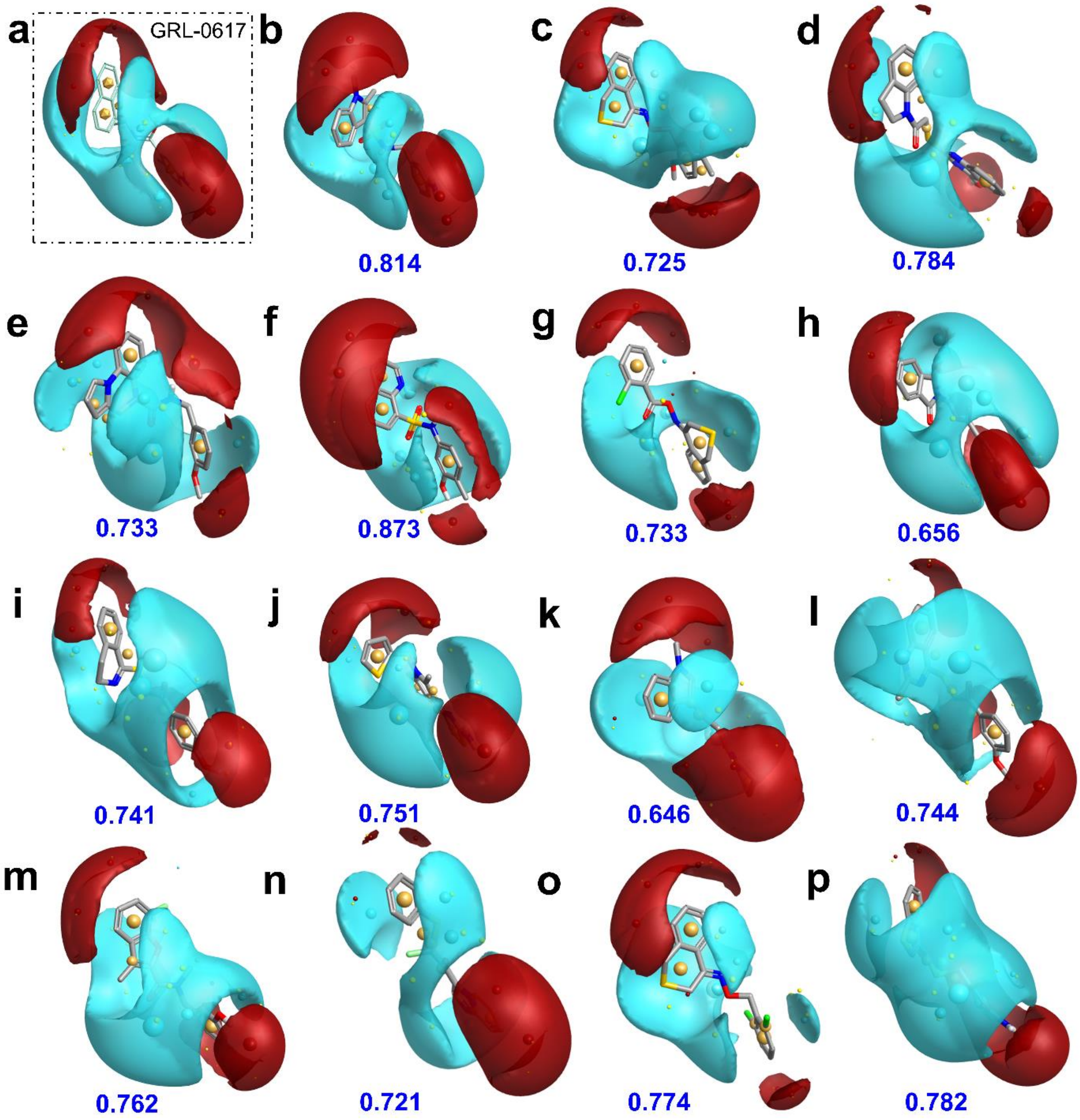

Figure S3. Hits' electrostatic field comparison to the reference (Forge results). Areas of positive electrostatic potential are shown in red. Areas of negative electrostatic potential are shown in cyan. Areas of hydrophobicity are shown as gold spheres. The compounds are shown as grey sticks. Field score is shown in blue font below each compound. PubChem CIDs: **a)** GRL-0617 (reference), **b)** 121589399, **c)** 5384279, **d)** 135457099, **e)** 46295452, **f)** 5183914, **g)** 5713704, **h)** 809521, **i)** 2822260, **j)** 4818469, **k)** 4433600, **l)** 4799709, **m)** 96565232, **n)** 2738453, **o)** 5713706, **p)** 2813610.

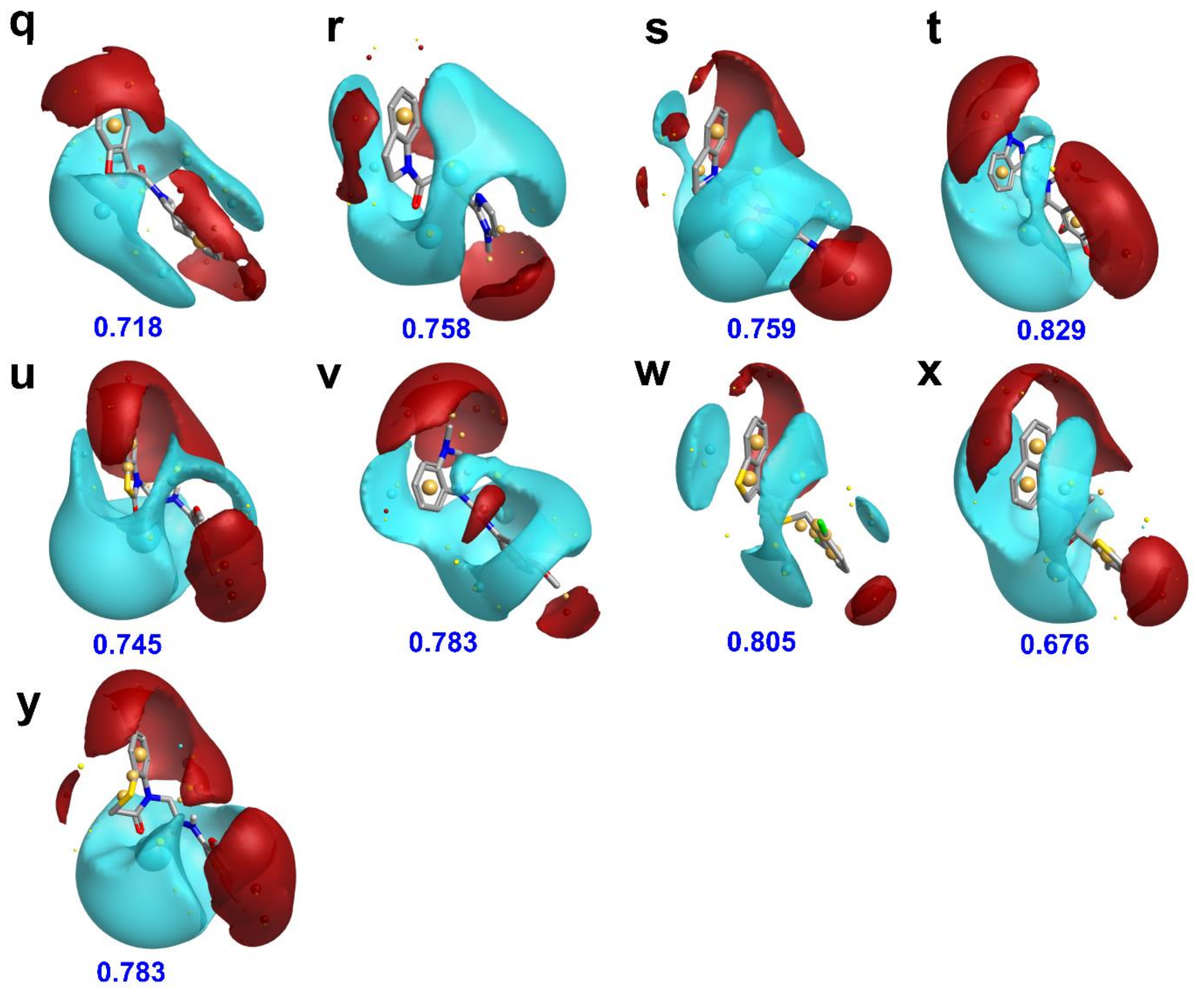

Figure S3 continued. PubChem CIDs: q) 5079298, **r)** 736576, **s)** 135431927, **t)** 53163980, **u)** 16043775, **v)** 46382930 **w)** 2732501 **x)** 128988277, **y)** 20878059.

Table S2: Vina results for docking control and 24 hits from ligand-based screening against PL^{pro}. The hits' PubChem CIDs are shown. Red boxes indicate the pose was not in the same binding site as GRL-0617. Where a compound's pose was not in the reference binding site, the binding affinity obtained in that run was excluded from the mean and standard error of the mean (SEM) calculations. The scores shown are that of the best ranking pose. The mean and SEM are calculated to 2 decimal places.

Compound ID	Run 1 ΔG (kcal/mol)	Run 2 ΔG (kcal/mol	Run 3 ΔG (kcal/mol	Run 4 ΔG (kcal/mol	Run 5 ΔG (kcal/mol	Mean	SEM
**GRL-0617**	**-8.8**	**-9.9**	**-9.9**	**-9.9**	**-9.9**	**-9.68**	**0.22**
**121589399**	-8.9	-9.0	-9.0	-8.6	-8.9	-8.88	0.07
**5384279**	-8.7	-8.7	-8.8	-8.5	-8.8	-8.70	0.05
**135457099**	-8.6	-8.6	-8.6	-8.6	-8.6	-8.60	0.00
**46295452**	-7.7	-8.3	-8.3	-8.9	-8.4	-8.48	0.14
**5183914**	-8.1	-8.6	-8.7	-8.1	-8.7	-8.44	0.14
**5713704**	-8.3	-8.4	-8.0	-7.9	-8.4	-8.20	0.10
**809521**	-7.9	-8.0	-7.9	-7.9	-8.0	-7.94	0.02
**2822260**	-7.9	-7.8	-7.7	-7.9	-7.8	-7.82	0.04
**4818469**	-7.4	-8.4	-7.2	-7.1	-8.3	-7.80	0.32
**4433600**	-8.1	-8.1	-7	-8.1	-7.7	-7.80	0.21
**4799709**	-7.3	-7.5	-7.8	-7.7	-7.8	-7.62	0.10
**96565232**	-7.5	-8.3	-7.4	-6.9	-7.7	-7.56	0.23
**2738453**	-6.7	-7.8	-8.0	-8.1	-6.8	-7.48	0.30
**5713706**	-7.9	-7.3	-7.9	-7.0	-7.2	-7.46	0.19
**2813610**	-7.5	-7.4	-7.5	-7.4	-7.4	-7.44	0.02
**5079298**	-7.2	-7.5	-7.2	-7.0	-7.1	-7.30	0.10
**736576**	-7.1	-7.2	-7.2	-7.0	-7.4	-7.18	0.07
**135431927**	-7.1	-7.2	-7.1	-7.2	-7.2	-7.18	0.03
**53163980**	-8.1	-7.5	-6.4	-7.3	-6.2	-7.10	0.35
**16043775**	-7.2	-7.2	-6.8	-6.8	-7.0	-7.00	0.09
**46382930**	-6.8	-6.9	-6.9	-6.9	-6.9	-6.88	0.02
**2732501**	-7.2	-6.8	-6.8	-6.8	-6.7	-6.86	0.09
**128988277**	-6.7	-6.5	-7.1	-7.1	-6.7	-6.82	0.12
**20878059**	-6.3	-6.5	-6.4	-6.4	-6.2	N/A	N/A

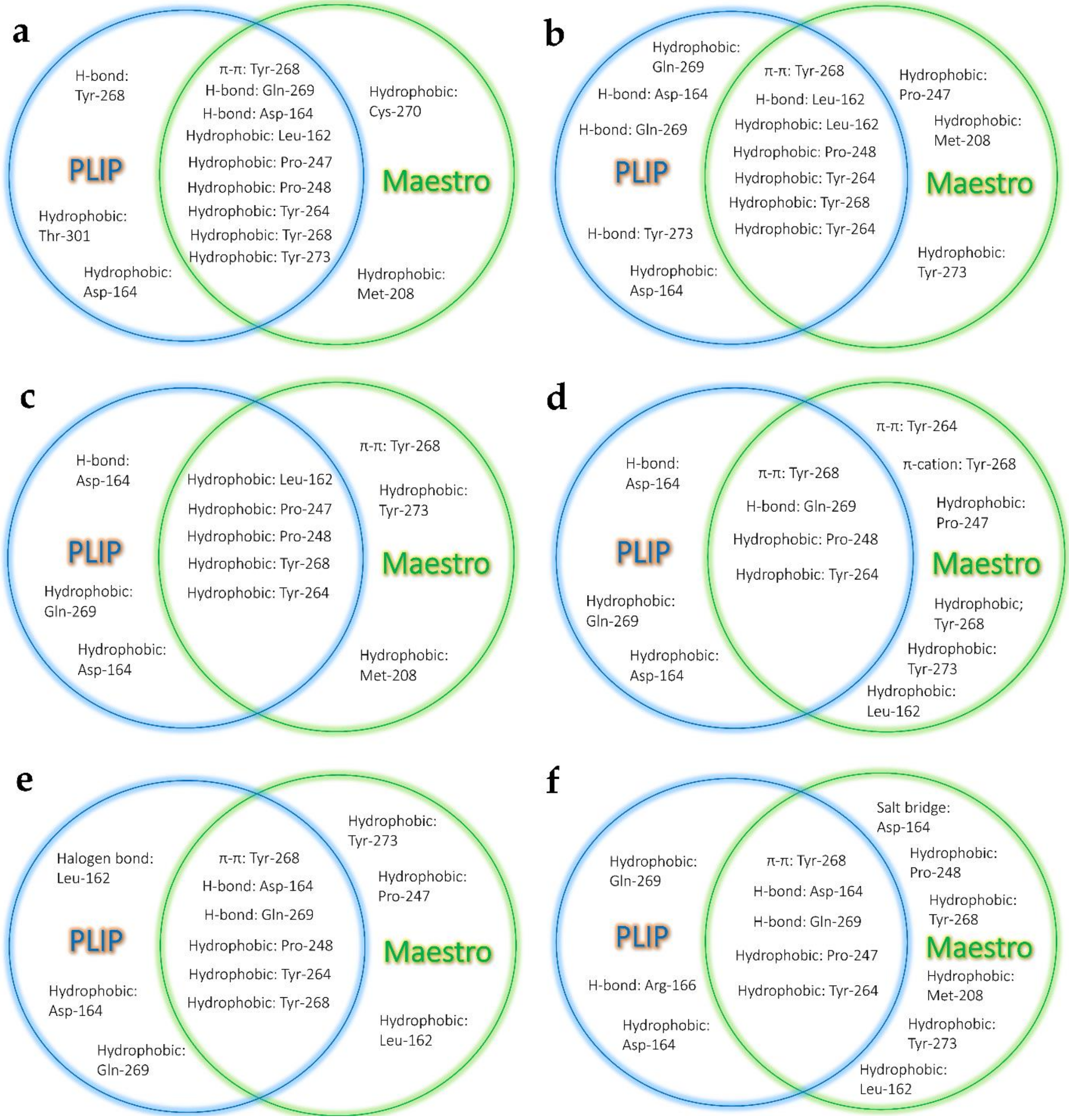

Figure S4: Venn diagrams showing the combination of PLIP and Maestro results for the reference and five selected hits. **a)** Combined results for reference GRL-0617; **b)** Combined results for the compound with PubChem CID 121589399; **c)** Combined results for the compound with PubChem CID 5384279; **d)** Combined results for the compound with PubChem CID 5183914; **e)** Combined results for the compound with PubChem CID 121558793; **f)** Combined results for the compound with PubChem CID 132344896.

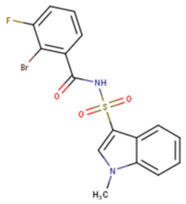
121558793

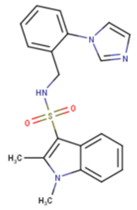
132344896

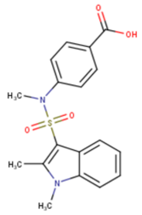
154858515

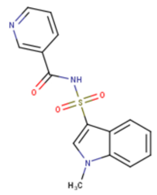
86814319

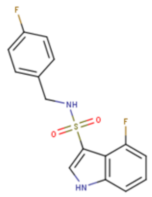
91648794

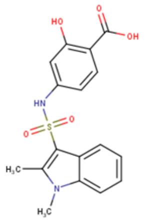
146091569

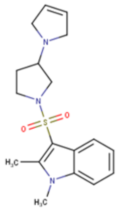
121527320

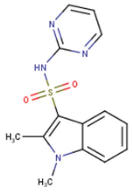
121527149

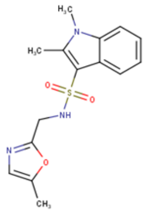
100636847

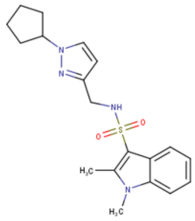
128313119

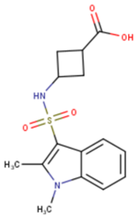
136557804

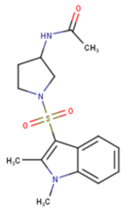
136548360

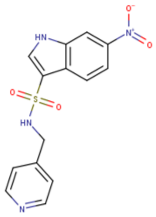
75437975

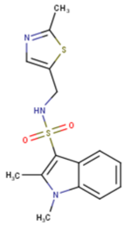
121589390

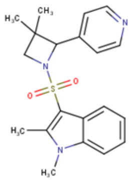
128503424

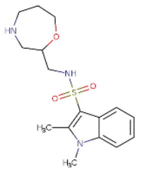
MolPort-046-428-607

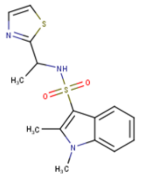
119040795

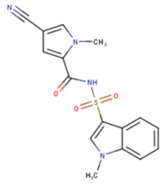
71967387

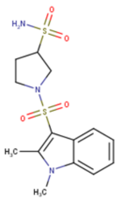
136541396

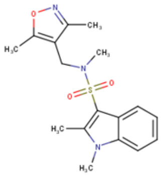
100636418

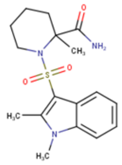
121527263

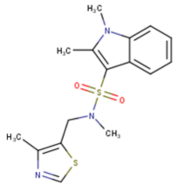
121555623

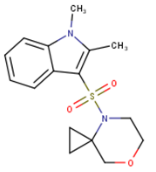
128313825

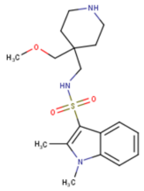
121708526

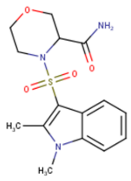
132374639

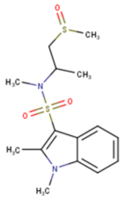
119052715

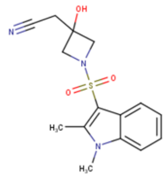
119052825

Figure S5. Two-dimensional structures of the analogues of compound 121589399. The structures were drawn using MarvinSketch. PubChem CIDs are shown below each structure. Where PubChem CID was not available, MolPort ID is shown.

Table S3: Commercial availability of 27 analogues of compound 121589399.

PubChem CID	MolPort ID	SMILES
**121558793**	MolPort-039-266-364	CN1C=C(C2=CC=CC=C21)S(=O)(=O)NC(=O)C3=C(C(=CC=C3)F)Br
**132344896**	MolPort-044-747-748	CC1=C(C2=CC=CC=C2N1C)S(=O)(=O)NCC3=CC=CC=C3N4C=CN=C4
**154858515**	MolPort-047-549-824	CC1=C(C2=CC=CC=C2N1C)S(=O)(=O)N(C)C3=CC=C(C=C3)C(=O)O
**86814319**	MolPort-035-387-863	CN1C=C(C2=CC=CC=C21)S(=O)(=O)NC(=O)C3=CN=CC=C3
**91648794**	MolPort-035-750-220	C1=CC2=C(C(=C1)F)C(=CN2)S(=O)(=O)NCC3=CC=C(C=C3)F
**146091569**	MolPort-046-898-600	CC1=C(C2=CC=CC=C2N1C)S(=O)(=O)NC3=CC(=C(C=C3)C(=O)O)O
**121527320**	MolPort-039-069-456	CC1=C(C2=CC=CC=C2N1C)S(=O)(=O)N3CCC(C3)N4CC=CC4
**121527149**	MolPort-039-069-284	CC1=C(C2=CC=CC=C2N1C)S(=O)(=O)NC3=NC=CC=N3
**100636847**	MolPort-038-995-536	CC1=CN=C(O1)CNS(=O)(=O)C2=C(N(C3=CC=CC=C32)C)C
**128313119**	MolPort-039-515-452	CC1=C(C2=CC=CC=C2N1C)S(=O)(=O)NCC3=NN(C=C3)C4CCCC4
**136557804**	MolPort-046-119-464	CC1=C(C2=CC=CC=C2N1C)S(=O)(=O)NC3CC(C3)C(=O)O
**136548360**	MolPort-046-108-923	CC1=C(C2=CC=CC=C2N1C)S(=O)(=O)N3CCC(C3)NC(=O)C
**75437975**	MolPort-028-783-276	C1=CC2=C(C=C1[N+](=O)[O-])NC=C2S(=O)(=O)NCC3=CC=NC=C3
**121589390**	MolPort-039-320-837	CC1=C(C2=CC=CC=C2N1C)S(=O)(=O)NCC3=CN=C(S3)C
**128503424**	MolPort-039-573-986	CC1=C(C2=CC=CC=C2N1C)S(=O)(=O)N3CC(C3C4=CC=NC=C4)(C)C
**N/A**	MolPort-046-428-607	Cc1c(c2ccccc2n1C)S(=O)(=O)NCC1CNCCCO1
**119040795**	MolPort-038-995-530	CC1=C(C2=CC=CC=C2N1C)S(=O)(=O)NC(C)C3=NC=CS3
**71967387**	MolPort-028-319-227	CN1C=C(C2=CC=CC=C21)S(=O)(=O)NC(=O)C3=CC(=CN3C)C#N
**136541396**	MolPort-046-101-487	CC1=C(C2=CC=CC=C2N1C)S(=O)(=O)N3CCC(C3)S(=O)(=O)N
**100636418**	MolPort-038-995-520	CC1=C(C2=CC=CC=C2N1C)S(=O)(=O)N(C)CC3=C(ON=C3C)C

Table S3 continued.

PubChem CID	MolPort ID	SMILES
**121527263**	MolPort-039-069-398	CC1=C(C2=CC=CC=C2N1C)S(=O)(=O)N3CCCCC3(C)C(=O)N
**121555623**	MolPort-039-251-349	CC1=C(SC=N1)CN(C)S(=O)(=O)C2=C(N(C3=CC=CC=C32)C)C
**128313825**	MolPort-039-518-191	CC1=C(C2=CC=CC=C2N1C)S(=O)(=O)N3CCOCC34CC4
**121708526**	MolPort-045-439-995	CC1=C(C2=CC=CC=C2N1C)S(=O)(=O)NCC3(CCNCC3)COC
**132374639**	MolPort-045-973-906	CC1=C(C2=CC=CC=C2N1C)S(=O)(=O)N3CCOCC3C(=O)N
**119052715**	MolPort-039-007-960	CC1=C(C2=CC=CC=C2N1C)S(=O)(=O)N(C)C(C)CS(=O)C
**119052825**	MolPort-039-008-068	CC1=C(C2=CC=CC=C2N1C)S(=O)(=O)N3CC(C3)(CC#N)O

Table S4. Vina results for docking control and 27 analogues of compound 121589399. Only the two best scoring analogues (highlighted in cream) proceeded to the next stage of analyses. The scores shown are that of the best ranking pose. The mean and standard error of the mean (SEM) is calculated to 2 decimal places.

Compound ID	Run 1 ΔG (kcal/mol)	Run 2 ΔG (kcal/mol	Run 3 ΔG (kcal/mol	Run 4 ΔG (kcal/mol	Run 5 ΔG (kcal/mol	Mean	SEM
**GRL-0617**	-9.90	-9.90	-9.90	-8.80	-9.90	-9.68	**0.22**
**121558793**	-9.40	-9.40	-9.40	-9.40	-9.40	-9.40	**0.00**
**132344896**	-9.18	-9.40	-9.30	-9.30	-9.10	-9.26	**0.05**
**154858515**	-8.40	-9.00	-9.00	-9.00	-8.90	-8.86	
**86814319**	-8.70	-8.70	-8.70	-8.70	-8.70	-8.70	
**91648794**	-8.70	-8.70	-8.60	-8.70	-8.70	-8.68	
**146091569**	-8.60	-8.70	-8.70	-8.60	-8.60	-8.64	
**121527320**	-8.70	-8.70	-8.50	-8.40	-8.70	-8.60	
**121527149**	-8.40	-8.50	-8.50	-8.40	-8.50	-8.46	
**100636847**	-8.20	-8.30	-8.20	-8.70	-8.30	-8.34	
**128313119**	-7.70	-8.20	-7.70	-9.30	-8.30	-8.24	
**136557804**	-8.20	-8.00	-8.20	-8.20	-8.00	-8.12	
**136548360**	-8.20	-8.20	-7.80	-8.20	-8.20	-8.12	
**75437975**	-8.10	-8.10	-8.10	-7.90	-8.30	-8.10	
**121589390**	-7.90	-8.00	-7.70	-7.90	-7.90	-7.88	
**128503424**	-6.80	-8.40	-8.00	-8.10	-7.10	-7.68	
**MolPort-046-428-607**	-8.00	-7.80	-7.70	-7.70	-7.70	-7.78	
**119040795**	-7.20	-8.00	-7.20	-7.20	-7.20	-7.36	
**71967387**	-7.40	-6.60	-7.20	-7.90	-7.90	-7.40	
**136541396**	-7.00	-6.80	-7.30	-6.80	-6.90	-6.96	
**100636418**	-7.10	-7.10	-6.50	-7.10	-7.10	-6.98	
**121527263**	-6.90	-6.90	-6.90	-6.80	-6.90	-6.88	
**121555623**	-6.90	-6.80	-6.90	-6.80	-6.20	-6.72	
**128313825**	-6.70	-6.70	-6.70	-6.70	-6.70	-6.70	
**121708526**	-6.50	-6.50	-6.60	-6.70	-6.70	-6.60	
**132374639**	-6.40	-6.40	-6.30	-6.90	-6.30	-6.46	
**119052715**	-6.40	-6.50	-6.20	-6.30	-6.40	-6.36	
**119052825**	-6.20	-6.50	-6.30	-6.30	-6.20	-6.30	

Table S5. Focused docking of the control and the five selected hits in AutoDock 4.2. The scores shown are that of the best ranking pose. The mean and standard error of the mean (SEM) is calculated to 2 decimal places.

Compound ID	Run 1 Δ G (kcal/mol)	Run 2 Δ G (kcal/mol)	Run 3 Δ G (kcal/mol)	Run 4 Δ G (kcal/mol)	Run 5 Δ G (kcal/mol)	Mean	SEM
**GRL-0617**	-9.49	-9.49	-9.28	-9.37	-9.52	**-9.43**	**0.05**
**121589399**	-8.16	-8.51	-8.19	-8.33	-8.20	**-8.28**	**0.06**
**5384279**	-8.24	-8.3	-8.28	-8.27	-8.27	**-8.27**	**0.01**
**5183914**	-8.09	-8.00	-8.22	-8.71	-8.09	**-8.22**	**0.13**
**121558793**	-9.19	-9.34	-9.04	-9.12	-9.15	**-9.17**	**0.05**
**132344896**	-8.63	-8.83	-8.75	-8.95	-8.63	**-8.76**	**0.06**

Table S6. Details of drug-likeness filters (rules) used in the SwissADME tool.

Rule	Details
Lipinski (Pfizer)	MW ≤ 500; MLOGP ≤ 4.15, N or O ≤ 10; NH or OH ≤ 5
Ghose (Amgen)	160 ≤ MW ≤ 480; -0.4 ≤ WLOGP ≤ 5.6; 40 ≤ MR ≤ 130; 20 ≤ atoms ≤ 70
Veber (GlaxoSmithKline)	Rotatable bonds ≤ 10; TPSA ≤ 140
Egan (Pharmacia)	WLOGP ≤ 5.88; TPSA ≤ 131.6
Muegge (Bayer)	200 ≤ MW ≤ 600; -2 ≤ XLOGP ≤ 5; TPSA ≤ 150; Num. rings ≤ 7; Num. carbons > 4; Num. heteroatoms > 1; Num. rotatable bonds ≤ 15; H-bond acc. ≤ 10; H-bond don. ≤5.
